# Large-scale land acquisitions exacerbate local farmland inequalities in Tanzania

**DOI:** 10.1073/pnas.2207398120

**Published:** 2023-07-31

**Authors:** Jonathan A. Sullivan, Cyrus Samii, Daniel G. Brown, Francis Moyo, Arun Agrawal

**Affiliations:** ^a^School of Geography, Development, and Environment, University of Arizona, Tucson, AZ 85721; ^b^School for Environment and Sustainability, University of Michigan, Ann Arbor, MI 48109; ^c^Wilf Family Department of Politics, New York University, New York City, NY 10012; ^d^School of Environmental and Forest Sciences, University of Washington, Seattle, WA 98195; ^e^School of Life Sciences and Bioengineering, Department of Sustainable Agriculture and Biodiversity Management, Nelson Mandela African Institution of Science and Technology, Arusha, Tanzania

**Keywords:** large-scale land acquisitions, inequality, land system science, causal inference

## Abstract

Land inequality stalls economic development, entrenches poverty, and is associated with environmental degradation. Yet, rigorous assessments of land-use interventions attend to inequality only rarely. A land inequality lens is especially important to understand how recent large-scale land acquisitions (LSLAs) affect smallholder and indigenous communities across as much as 100 million hectares around the world. This paper studies inequalities in land assets, specifically landholdings and farm size, to derive insights into the distributional outcomes of LSLAs. Using a household survey covering four pairs of land acquisition and control sites in Tanzania, we use a quasi-experimental design to characterize changes in land inequality and subsequent impacts on well-being. We find convincing evidence that LSLAs in Tanzania lead to both reduced landholdings and greater farmland inequality among smallholders. Households in proximity to LSLAs are associated with 21.1% (*P* = 0.02) smaller landholdings while evidence, although insignificant, is suggestive that farm sizes are also declining. Aggregate estimates, however, hide that households in the bottom quartiles of farm size suffer the brunt of landlessness and land loss induced by LSLAs that combine to generate greater farmland inequality. Additional analyses find that land inequality is not offset by improvements in other livelihood dimensions, rather farm size decreases among households near LSLAs are associated with no income improvements, lower wealth, increased poverty, and higher food insecurity. The results demonstrate that without explicit consideration of distributional outcomes, land-use policies can systematically reinforce existing inequalities.

## Significance

Land is a principal component of sustainability but inequalities in land use and control can adversely affect both people and the environment. It is unclear to what extent land-use policies contribute to growing land inequality. Here, we study the dynamics of land inequality in the context of large-scale land acquisitions in Tanzania that aim to increase commercial crop production. We show that land acquisitions exacerbate farmland inequality in nearby villages while farmland distribution elsewhere remains unchanged. The resulting inequalities are not offset by improvements in other dimensions of well-being, rather affected households are left worse off. Our study demonstrates that without explicit consideration of distributional outcomes, land-use interventions can systematically generate greater inequalities with important consequences for livelihoods and development.

Land is an essential component of rural livelihoods for more than 380 million households but is among the many resources that are inequitably distributed (1, 2). The top 10% of rural populations capture 60% of agricultural land value, whereas the bottom half capture only 3% (3). Beyond normative arguments for more equitable distributions of land, a substantial literature on land inequality reveals its negative influence on human–environment systems. Higher levels of land inequality are associated with lower economic development (4), higher rates of forest loss (5), and distortion of political institutions that favor elites (6). Despite the recognized trends of worsening land concentration and its importance to human–environment systems, our understanding of how land-use interventions affect land inequality and distribution remains poorly understood (7). Here, we use quantitative data to assess how one of the largest disturbances to who owns and uses land—large-scale land acquisitions (LSLAs)—reshuffles local land control, changes land inequality, and subsequently impacts well-being within nearby communities.

In the early 2000s, a confluence of food price volatility, looming land scarcity, and increasing land values raised the interest of private investors in farm and forest land acquisitions (8–10). As a result, LSLAs surged globally, with at least 63 million hectares of agricultural land purchased or leased by investors since 2000 (11). Within this global trend, sub-Saharan Africa is a hotspot of land investments where 25% of concluded LSLAs occurred since 2000, often in agricultural areas already in use by smallholder farmers (12, 13). As a result, LSLAs fundamentally involve the transfer of land rights, generally from smallholder farmers or pastoralists to private companies, with dispossession or displacement being commonplace (14, 15).

The byproducts of such large-scale changes to tenure are hotly debated. Some raise serious concerns for rural livelihoods and the environment, for example, showing that LSLAs heighten food insecurity (16), provide few employment opportunities (17), increase water scarcity (18), and accelerate forest loss and carbon emissions (19, 20). On the other hand, governments in many lower and middle-income countries pursued investments under the logic that LSLAs would bring new capital and technology to modernize and improve agricultural productivity and development (21, 22). LSLAs may boost rural economies in several ways including improved market access through contract farming programs (23), spillovers of new agricultural technology, (24) and indirect benefits to nonfarm employment (25). Such pro-investment arguments are based on the concept of agricultural transformation where larger, more efficient farms improve their productivity while small-scale, inefficient farmers move to better, off-farm opportunities leading to welfare improvements (26). Arguments that the redistribution of large swathes of land will promote agricultural transformation, however, stand in contrast to literature on land concentration and land reform.

Currently, examinations of LSLAs lack a recognition of how greater land concentration may challenge the development narratives of investors or host governments. Three strands of literature on land concentration and land reform stand out. First, where land is more highly concentrated, agricultural productivity and rural incomes are suppressed (27, 28). This is consistent with studies on the spillover effects of LSLAs that find null or negative effects on crop yields (24, 29, 30). A second set of literature on land inequality argues that large landowners tend to oppose public goods and human capital investments, such as education, that would increase rural wages and off-farm opportunities removing important labor sources (31). In connection with these theories are studies that find slower economic growth and lower development outcomes in countries with higher land inequality (32). Finally, literature on land reform argues large landowners can distort local politics through patron–client relationships, embedding power and making land inequality intractable (6). Indeed, we see that LSLAs are resistant to demands of social mobilizations with disconcerting levels of repression, violent targeting, and criminalization of local activists (33). Thus, LSLAs can influence human–environment systems, well-being, and power structures by reshaping local land inequality, a primary effect of LSLAs that remains unexplored.

To date, studies on LSLAs emphasize the loss of land assets as a principal outcome but provide little counterfactual evidence and do not focus on land inequality. LSLAs regularly occur in areas under cultivation and coincide with land loss confirmed in studies using household and remote sensing analyses, for example, in Ghana (34) Mozambique, (35, 36) and Ethiopia (37). Several qualitative case studies suggest that land loss is uneven and vulnerable populations are systematically excluded through socio-political processes. For example, large landowners directly negotiated with investors in Laos to maintain their land rights in exchange for selling their harvest to a newly established LSLA (38). Similarly, large landholders in Tanzania face fewer barriers in accessing new business opportunities, such as contract farming, creating greater social differentiation (39, 40). Despite the importance of land inequality and the potential for LSLAs to reshape land concentration, to our knowledge, there are no quantitative or counterfactual analyses that examine the distributional impacts of LSLAs on local land assets or its mediating effects on well-being.

To address this gap, the objective of this paper is to understand the relationship between LSLAs, land inequality, and household well-being. Specifically, we answer the following research questions: i) Do LSLAs exacerbate or ameliorate inequality in farm size, landholdings, and land value? ii) How do household baseline characteristics of land, labor, or income explain mechanisms of distributional change in land assets? iii) What are the implications of LSLA-driven changes in farmland distribution for household well-being as measured by income, wealth, poverty, and food insecurity? We elect to use farm size and landholdings as two measures of land assets, as they serve distinct socio-economic functions and reveal mechanisms at play. For example, farm size is associated with agricultural incomes and its distribution can influence local food security (41). Importantly, farm size is frequently used to assess land inequality given its importance to rural livelihoods (3, 4, 27). Landholdings, on the other hand, are a component of household wealth that can appreciate or be transformed into financial, human, or productive forms of capital (42, 43). Together, farm size and landholdings play central but unique roles in understanding human–environment systems (44).

**Fig. 1.**
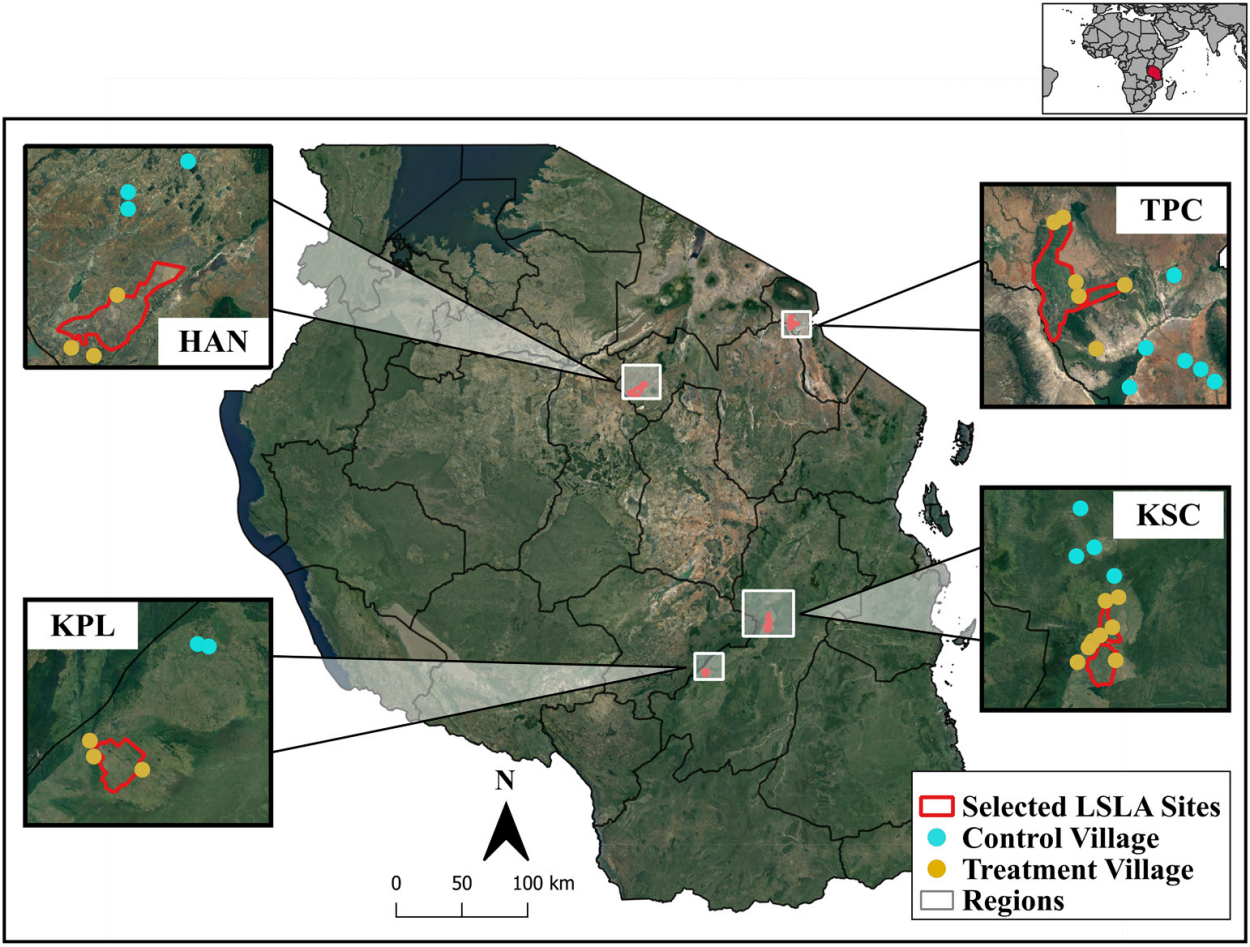
Our selected LSLA sites in Tanzania cover a range of geographic contexts including the dry northern highlands and subhumid tropics in the south. For each of the selected sites—Kilombero Plantation Ltd. (KPL), Hanang Wheat Complex (HAN), Tanganyika Plantation Co. (TPC), and Kilombero Sugar Company (KSC)—we selected LSLA (n = 20) and control (n = 15) villages in which we implemented our household survey in 2018 and 2019. Figure previously published in Sullivan et al. (30). Base map—Google Earth.

We examined our research questions across four pairs of LSLA and control sites in Tanzania using secondary datasets and our own household survey collected in 2018 comprising 994 households across 35 villages (Fig. 1 and Table 1). Tanzania is a hotspot of land acquisitions and emblematic of development debates surrounding LSLAs. In particular, international and national initiatives in Tanzania seek to improve agricultural economies with private investment, including the support of LSLAs, where 73 to 82% of the population relies on agriculture for income and food (45, 46). Together these policies, including the New Alliance for Food Security and Nutrition, the Southern Agricultural Growth Corridor of Tanzania (47), Kilimo Kwanza (Agriculture First), and Big Results Now, attracted $1 billion of agricultural investments that coincided with an estimated 42 LSLAs totaling ~350,000 ha (11, 48).

**Table 1.** Summary of the four selected LSLAs in Tanzania

Site name	Company	Acquisition year	Region	LSLA size (ha)	Main crop	Potential farmland (hectares/household)
Kilombero Sugar Company (KSC)	Illovo Sugar Ltd. (South Africa)	2000	Morogoro	12,298	Sugarcane	2.6
Kilombero Plantation Ltd. (KPL)	Agrica Ltd. (United Kingdom)	2008	Morogoro	5,158	Rice	2.6
Tanganyika Plantation Company (TPC)	Alteo Ltd. (Maritius)	2000	Kilimanjaro	10,349	Sugarcane	0.9
Hanang Wheat Complex (HAN)	Ngano Ltd. (Kenya)	2005	Manyara	17,790	Wheat	1.5

In this study, we use a quasi-experimental design in combination with linear and quantile regression to show that LSLA-proximate villages are associated with lower landholdings and greater farmland inequality, when compared with similar but more distant households and villages. In addition, using a mediation analysis we find that changes in farmland distribution are associated with declines in well-being. Our focus on inequality and its connections with well-being carry important implications for how land tenure changes associated with LSLAs may systematically exclude certain populations with ramifications for agricultural development.

## Overview of Study Area and Methods

From a known set of 25 LSLAs with geolocation data, we selected four LSLA sites that prior work shows are representative of the larger set (30). The selected LSLA sites include i) the Kilombero Plantation Ltd—a rice plantation, ii) the Kilombero Sugar Company—a sugar cane plantation with approximately 8,500 farmers engaged in contract-farming (40), iii) the Tanganyika Plantation Company—another sugarcane plantation that relies on irrigation, and iv) the Hanang Wheat Complex—a remote wheat plantation in Manyara (Table 1).

Land tenure changes associated with the selected LSLAs involve either the leasing of public land or redesignating village land—a form of legally recognized customary land—to then be leased. While these are separate legal processes depending on the prior *de jure* tenure, commonalities of dispossession and displacement exist. In the case of leasing public land, even where *de jure* rights are claimed by the state, multiple *de facto* claims to land exist in concession areas that were inactive for decades with unclear boundaries (49). Where village land is redesignated, numerous procedures and consultations with village authorities are required that, in theory, protect land rights. Previous studies, however, show that in practice, village authorities can agree willingly or not, receive varying degrees of information, and central agencies oversee technical mapping or land transfer procedures that can create conflicts (50). Regardless of the exact form of tenure change, the state and investors use various legal mechanisms including rhetoric of “idle lands” to access occupied, rural land that can result in dispossession and redistribution (51, 52).

Using our household survey and a matched, quasi-experimental design, we estimate the average and distributional effects of LSLAs on household land assets with cross-sectional and difference-in-differences estimators. Our analysis compares post-acquisition land assets between treatment households—those residing in villages within 5 kilometers of LSLAs and where LSLAs induced land tenure change—to matched, control households—those far from LSLAs but with similar socio-demographic characteristics (53, 54). To investigate the influence of LSLAs on land value, we use the National Panel Survey in 2009 and 2013. Using cross-sectional estimates, we examine the associations between household land value, distance from LSLA, and land asset size to reveal heterogeneous effects on land markets. We investigate causal mechanisms contributing to observed LSLA impacts on land distribution using flexible interaction models (55). Finally, using mediation analysis, we link changes in farmland distribution with measures of well-being including total income, a wealth asset index, a multidimensional poverty index, and food insecurity (43, 56, 57).

## Results

### Impact of LSLAs on Land Assets and Markets.

#### Average household effects.

**Fig. 2.**
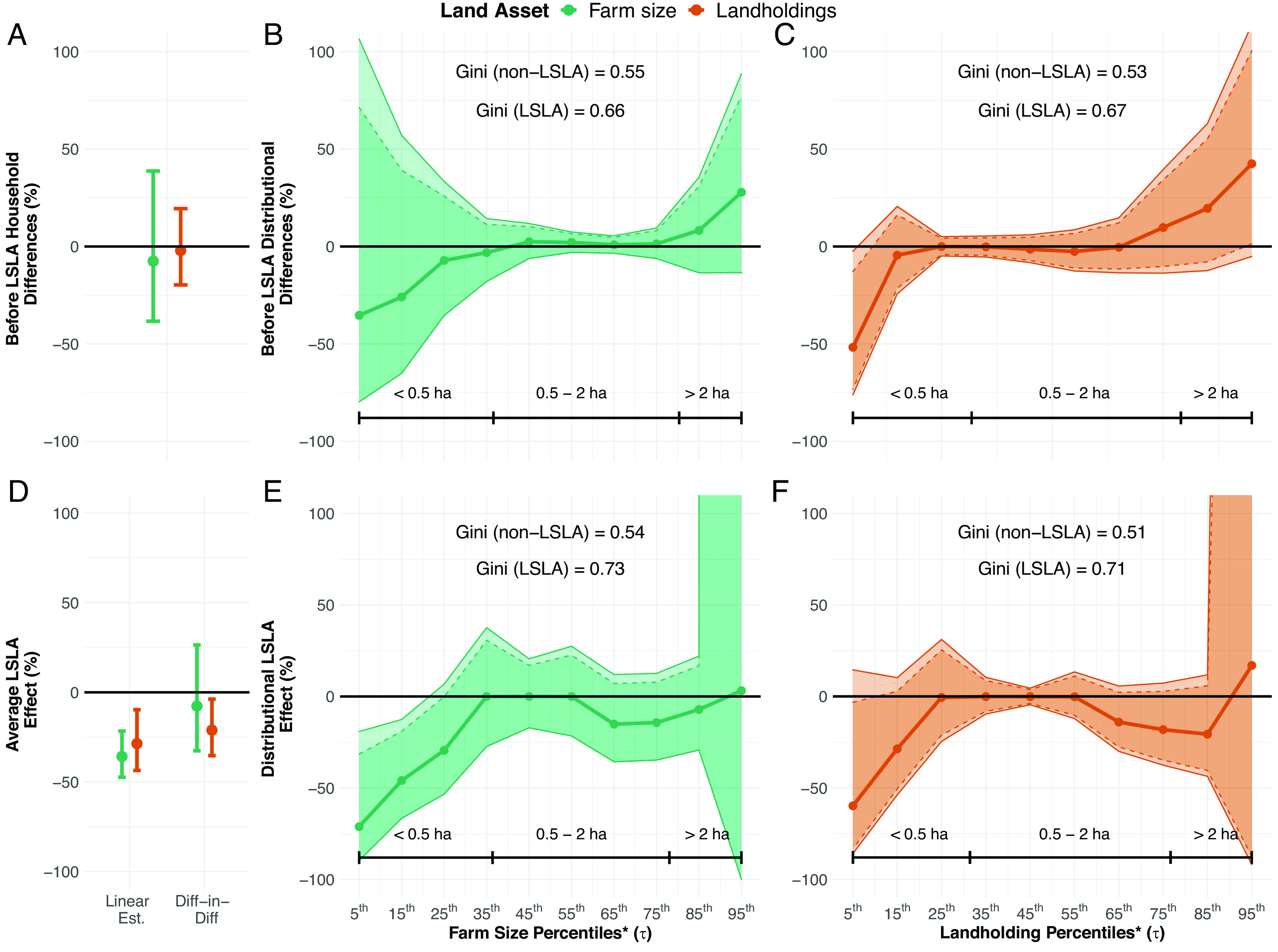
Estimates of the average and distributional effects of LSLAs on farm size and landholdings. We provide estimates for before-LSLA impacts on land assets for (*A*) average outcomes; (*B*) distributional estimates for farm size; and (*C*) distributional estimates for landholdings. After-LSLA influence on land assets are estimated for (*D*) linear and difference-in-differences average estimates; (*E*) distributional estimates for farm size and (*F*) distributional estimates for landholdings. Dark shaded areas around point mean estimates are the 90% CIs and light shaded areas are the 95% CIs. * = land asset outcomes that are log transformed. Log-transformed average treatment effect (ATE) and quantile treatment effect (QTE) estimates are converted to percentages using the equation: $\mathrm{percent}\;\mathrm{effect}\;\mathrm{size}=\left(\exp\left(\beta \right)-1\right)*100$.

Following LSLAs, treatment households had smaller landholdings than control households and evidence is suggestive that farm sizes also declined. In our cross-sectional estimates, differences are significant both statistically and in magnitude, with estimated farm sizes and landholdings being 35.8% (*P* < 0.001) and 28.6% (*P* < 0.01) smaller in treatment households, respectively (Fig. 2*D* and *SI Appendix*, Table S1). Estimates of baseline land assets find no substantial differences between treatment and control households, providing evidence that post-acquisition differences arise due to LSLAs (Fig. 2*A* and *SI Appendix*, Table S1). Our estimates use a reweighted sample that is balanced across all covariates also included in linear specifications (*SI Appendix*, section 1.3). To check the robustness of our average estimates, we evaluated changes in land assets using a matched, difference-in-differences design. Household landholdings decreased by 21.1% (*P* = 0.02) among households in LSLA villages as compared to non-LSLA households, and farm size estimates indicate a 7.7% decrease, but the effect was not statistically significant (Fig. 2*D* and *SI Appendix*, Table S1). However, the cross-sectional estimator controlling for pretreatment outcomes and the difference-in-differences estimator have a bracketing relationship, suggesting that our estimate for farm size is negative (58, 59). Our average estimates demonstrate declining land assets among treatment households but, like all linear estimates, mask information related to heterogeneity in these effects.

#### Distributional effects.

Based on quantile regression results, we find that inequality in farmland distribution increased (Fig. 2 *B*–*E*) but despite average declines in landholdings, we find no significant effects on landholding inequality (Fig. 2 *C*–*F*). We find land-poor, treatment households (< 0.5 ha of land) between the 5th and 25th percentiles have smaller farm sizes than control households within equivalent percentiles. Among land-poor households, the largest negative effects are found at the fifth percentile, where treatment households have 71% (*P* = 0.02) smaller farm sizes than control households within the same percentile. Effect sizes dissipate but remain significant at the 15th percentile (estimate: −46%, *P* = 0.01) while further converging toward zero at the 25th percentile (estimate: −29%, *P* = 0.1) (Fig. 2*E*). Finally, large farms in the 95th percentile who cultivate at least 6.5-ha have 3.2% greater farm sizes than control households, although high variance in estimates obscures any statistically significant results (Fig. 2*E* and *SI Appendix*, Table S2).

For landholdings, we only find significant results at the fifth quantile with 59% (*P* = 0.09) smaller landholdings among treatment household. However, this pattern was also present prior to LSLAs and thus we cannot conclude that increases in landholding inequality occurred. While negative but insignificant point estimates are found at the 15th percentile suggesting a pattern of increasing inequality, we also see similar estimates at the 65th, 75th, and 85th percentiles (*P* ranging from 0.13 to 0.19). A worsening Gini coefficient among treatment households is another indicator that landholding inequality may be worsening; however, we cannot statistically test for differences in this aggregate measure (Fig. 2*F* and *SI Appendix*, Table S3).

We check for differences between treatment and control households in baseline land assets and find no significant effects across percentiles, except for landholdings within the fifth percentile (Fig. 2 *B* and *C* and *SI Appendix*, Tables S4 and S5). Moreover, we use preacquisition land assets in weighting and regression specifications thus controlling for baseline differences where they exist. Finally, our covariate control strategy relies on households’ recall of preacquisition land assets that can introduce nonclassical errors despite our use of best survey practices (60). We check the robustness of our results against recall bias using a smaller round of surveys in 2019 (n = 172). We find no systematic patterns of recall error by treatment status suggesting that recall bias does not influence our effect size estimates (*SI Appendix*, section 1.6).

#### Land value.

Land value is a vital component of land inequality, insofar as land relates to dimensions of wealth. Using the National Panel Survey in 2009 and 2013, we find no evidence of inequality in land values across land asset size (*SI Appendix*, Table S6). Thus, the major determinant of land inequality in our study area remains differences in land area as statistically confirmed in the case of farm size and descriptively suggested in the case of landholdings. We do, however, find a significant effect of increasing land values the closer a household resides to an LSLA (*SI Appendix*, Fig. S1). Effects sizes are substantial with household farm size and landholding values declining an estimated 13% and 12%, respectively, for households just 10 kms from LSLAs. While associations between LSLAs and higher land prices are not causal, this suggests a key mechanism of high levels of land competition that contributes to differences in farmland inequality. Semistructured interviews further strengthen this finding where poor land access was cited due to economic barriers including the costs of land.

### Household Mechanisms of Land Asset Change.

**Fig. 3.**
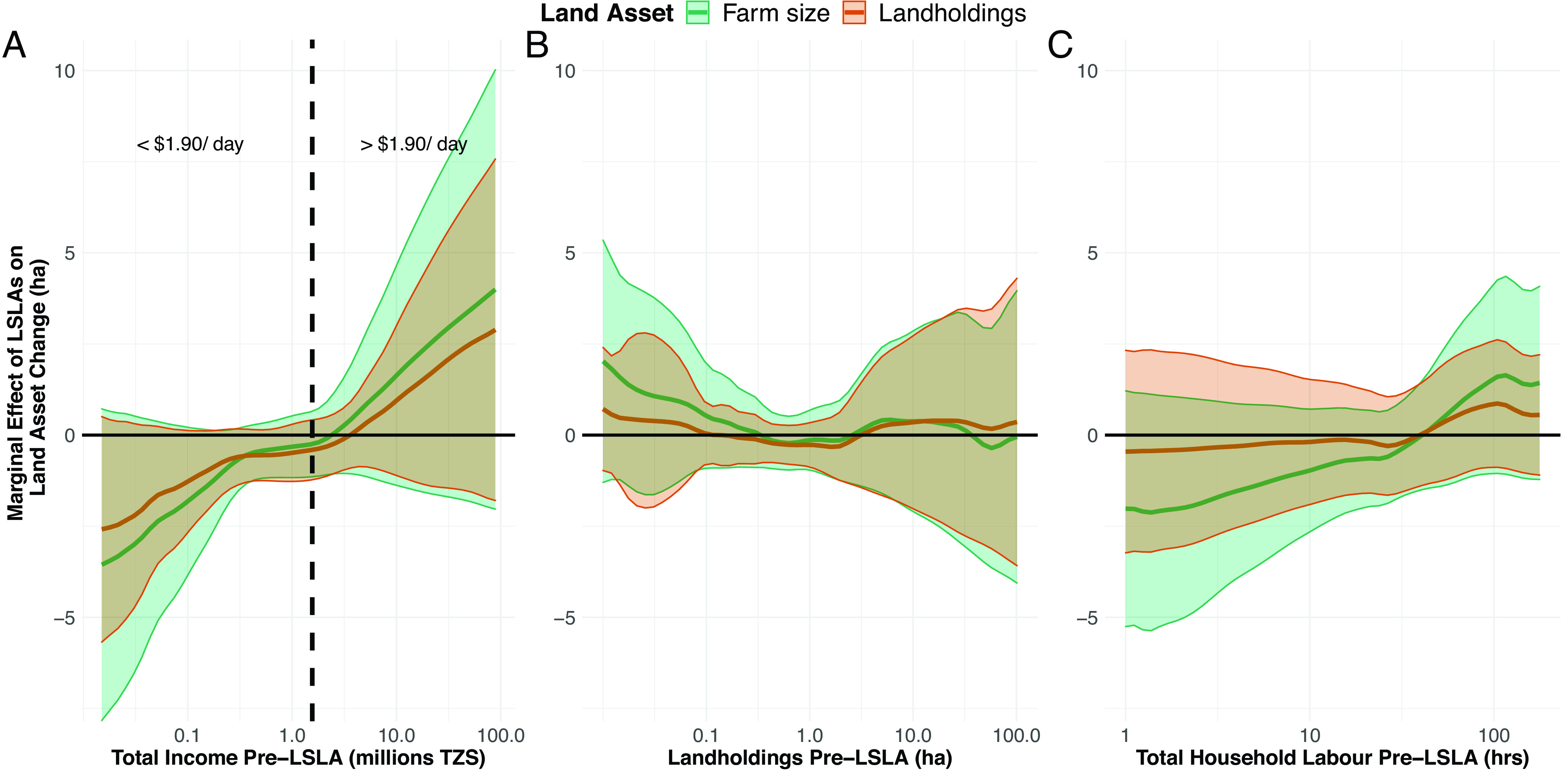
Changes in land assets as explained by household characteristics. Moderation effects of household baseline conditions in (*A*) income, (*B*) landholdings (wealth), and (*C*) total available labor on land asset change. Shaded areas around point mean estimates are the 95% CIs.

We investigated how baseline household income, land assets, and labor modified the effect of LSLAs on land distributions using flexible and linear interaction models (55). We find that the effects of LSLAs on land loss worsen with declining incomes (Fig. 3*A* and *SI Appendix*, Table S7). Based on point estimates, households below the poverty line ($1.90 per day) decreased landholdings between 0.4 and 2.6 ha and farm sizes between 0.3 and 3.6 ha, with the greatest reductions for households at lower incomes. Above the poverty line, households exhibit a mixed response with higher levels of income associated with increases in land assets. Baseline household land assets and total labor exhibit no clear pattern of moderation on the effect of LSLAs on household land asset change (Fig. 3 *B* and *C* and *SI Appendix*, Tables S8 and S9). The CIs for the income-specific effects include zero and linear specifications do not yield significant estimates (landholding *P* = 0.23; farm size *P* = 0.18). Nevertheless, our collective evidence suggests that income offers the means for households to increase their land assets or, conversely, is a barrier to some households to maintaining their presence in or (re)entering increasingly expensive land markets.

We explored alternative household-level explanations of changing land distributions including differences in land rental activity, out-migration, and in-migration across treatment and control households. Using Pearson correlation coefficients, we find landholdings are positively correlated with renting-out land while farm size is positively correlated with renting-in land (*SI Appendix*, Fig. S2). However, we find the same patterns of land rental activity among treatment and control households and conclude that land rental markets are not a key mechanism explaining our results. We also find similar levels of out-migration and reasons for migrating among treatment and control households (*SI Appendix*, Figs. S3 and S4). However, we lack data on out-migrants that relocate between baseline and survey years and our results relate only to those remaining within our surveyed villages. Finally, from household rosters collected during fieldwork, we observe that treatment villages have populations 55% higher than control villages (*SI Appendix*, section 1.1). After controlling baseline population levels in our estimates, this suggests that in-migration is a likely mechanism driving our results not captured in our household surveys.

### Impact of Farmland Inequality on Well-Being.

**Fig. 4.**
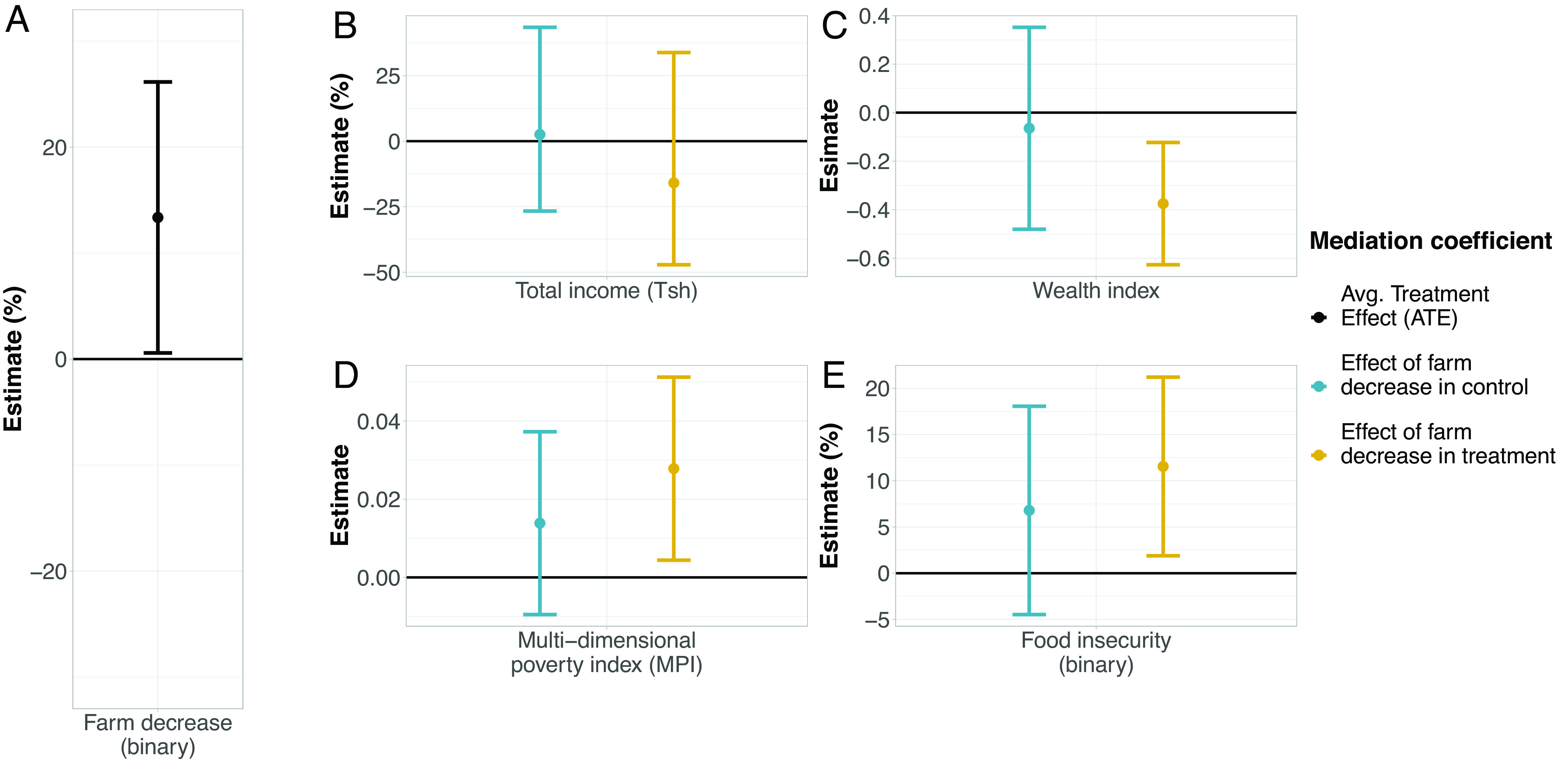
Effect of LSLAs on household well-being as mediated by farmland decreases. Using a mediation analysis, we estimate (*A*) the effect of LSLAs on the likelihood of household farmland decreases and the effect of farmland decreases on household (*B*) total income, (*C*) wealth using an asset index, (*D*) poverty using a multi-dimensional index, and (*E*) food insecurity. Full results of the mediation analysis can be found in *SI Appendix*, Table S10.

We investigated the impact of household decreases in farmland, a primary mechanism by which we observe increasing farmland inequality surrounding LSLAs (*SI Appendix*, Fig. S5), on several measures of well-being using a mediation analysis. This analysis involves, first, estimating the effect of LSLAs on farmland decreases, and, second, estimating the effect of such decreases on welfare measures, controlling for treatment status (56). Consistent with the results above, treatment households are 13.3% more likely to experience decreases in farmland. These decreases, in turn, predominantly have negative impacts on well-being (Fig. 4*A* and *SI Appendix*, Table S10). The effects of farm decreases among treatment households include lower wealth by 0.30 control group SDs (estimate = 0.37; *P* < 0.01), higher poverty by 0.21 control group SDs (estimate = 0.03; *P* < 0.1), and a 12% (*P* < 0.1) increased likelihood of experiencing food insufficiency (Fig. 4 C, D, and E). In the case of total income, we find no significant results suggesting treatment households are no better or worse off in this dimension of well-being (Fig. 4*B*). We observe no significant effects among control households suggesting unique and negative influences of LSLA-driven land inequality on household well-being. A likely contributing factor to declines in well-being is low levels of compensation, cash or in-kind, across all LSLAs (*SI Appendix*, Fig. S6). As before, our results on well-being are limited to households remaining in surveyed villages and we lack data on the well-being status of out-migrants.

## Discussion

We find the implementation of LSLAs in Tanzania is associated with smaller landholdings and likely farm sizes, exacerbated farmland inequality, and lower forms of well-being among dispossessed households. Our results have three broad implications for our understanding of how LSLAs relate to land tenure change and their significance for agricultural development. First, these findings provide important empirical evidence in support of theoretical arguments about land governance and LSLAs. They raise attention to the issue that rural smallholders are systematically dispossessed by LSLAs where investors, ministry officials, and local elites can shape negotiations over land rights at multiple levels. It is well established that LSLAs occur in lands under customary tenure, (14, 15) where their weak legal status or declaration as “idle” land results in the loss of land rights for many (51, 52). However, residing within customary lands does not make all its inhabitants equally vulnerable to dispossession. Rather, the negotiation of land rights for LSLAs are influenced by patron relations (39), preferential access to local elites in acquisition processes (38), undemocratic procedures at the community level (51), and low transparency and information from centralized ministries (50) that altogether can exacerbate existing inequalities. Remarkably, we find this result in Tanzania where protection and security of customary land rights is arguably the most advanced in sub-Saharan Africa. While customary tenure is legally recognized in Tanzania, it does not make it impervious to government interests in centralizing land control (21). Our study takes these theories and arguments that LSLAs tend to consolidate existing processes of differentiation and connects them to observed changes in distributional outcomes.

Second, the linkage between LSLAs and land value is an important mechanism in explaining land inequality and outcomes within human–environment systems. Prior work on LSLAs is primarily focused on the institutional processes that influence dispossession, livelihoods, and the environment with no examples, to our knowledge, of how LSLAs influence land markets (14, 15). The positive association between LSLAs and land values means that dispossessed smallholder farmers with less or no farmland face relatively high economic barriers to reenter land markets, making land inequality durable. This effect is exacerbated in the context of low compensation of displaced households that we find in our analysis and commonly reported in literature (14). While our analysis is unable to uncover the causal relationship between LSLAs and land value, the implications for explaining outcomes in human–environment systems remain important and is currently underexplored.

Finally, contrary to common narratives that LSLAs induce agricultural development amidst land redistribution, we find that households with declining farmland assets, whether voluntary or forced, do not recover to baseline levels of well-being. Land concentration can be a feature of agricultural transformation but can diminish well-being where linkages to nonfarm economies are lacking (26). There is significant evidence that LSLAs dispossess small-scale producers while providing little to no compensation (14). Additionally, LSLAs can result in lower levels of local employment and commonly provide only seasonal employment generating few off-farm opportunities (17). Together, these conditions can create barriers for dispossessed or landless households to convert farmland to capital or seek employment elsewhere that would assist in transitioning livelihoods. Our results concur with these studies where we find worsened socio-economic conditions and provide strong evidence this is linked to growing land inequality.

Our study contributes to improved methods for examining LSLA effects on agrarian structure using counterfactual analysis and quantile regression to identify causal and distributional effects of LSLAs. Notwithstanding the advances made here, our survey and analysis contain some limitations. First, our survey relies on recall data that, despite best survey practices such as event anchoring, can introduce noise and nonclassical errors into our analysis (60). To the extent possible we assess recall error across treatment and control households, not commonly found in causal inference studies on natural resource policies, finding no systematic patterns of bias. Second, our household survey does not account for urban-based, absentee landowners, nor for in-migration, and thus is limited in scope to land asset impacts on nearby residents. We also do not account for out-migrants and thus our analysis of socio-economic impacts is limited, especially if out-migrants receive greater forms of compensation.

Debates on the agricultural and development impacts of LSLAs are split between critics who decry human rights abuses and environmental damage versus proponents that defend the ability of private investments to generate employment and benefits. By focusing on the distributional impacts of LSLAs using rigorous causal inference methods and quantile regression, our study highlights how LSLAs are potential sites of polarization and differentiation. Similar arguments are found in case studies but typically lost in quantitative studies that characterize average effects. Given the critical effects that land inequality and concentration can have on development, the environment, and governance opportunities, understanding changes in agrarian structure surrounding LSLAs is of key importance. Future studies should be attentive to how LSLAs influence land concentration, land values, and how land concentration in turn affects human–environment outcomes.

## Methods

### Quasi-experimental Design and Sampling.

Our study uses a quasi-experimental design increasingly used in natural resource studies (61). The main feature of our research design identifies households directly affected by LSLAs (treatment) and those unaffected but with similar socio-economic and geographic characteristics (control). We define villages as “treated,” and equivalently households within those villages, that are within 5 kilometers of a selected LSLA boundary and where i) tenure change occurred or ii) a village shares a boundary with the LSLA. To select plausible counterfactuals, we identify control areas with similar socio-ecological characteristics, and within those areas, we enumerate all eligible control villages. From the set of all eligible treatment and control villages, we randomly selected 35 villages and subsequently 1,003 households to survey using a stratified random sample (*SI Appendix*, Table S11). More details on the site selection, village and household sampling, survey design, and data processing are available in *SI Appendix*, section 1.1.

### Land Asset and Well-Being Outcomes.

Our outcome variables include farm size, measured as cultivated area by household, and total landholdings. Farm size may include rented land for the purposes of cultivation. Landholdings include those under a Certificate of Customary Right of Occupancy, a substitute for titles on village land, as well as those recognized as customary holdings. Collectively we refer to these outcomes as land assets and our regression models use log-transformed land assets with the addition of a small constant to handle households with no land assets (i.e., log[land asset + 0.01]). We evaluate baseline, post-acquisition (2018), and change outcomes. The land asset change outcome is calculated as the difference between post- and baseline household assets with no transformation.

Our analysis of well-being includes four outcomes: total income, wealth, poverty, and food insecurity. Total income represents both farm income and nonfarm income sources such as wages, business profits, and land rental reported by households in our survey. Household wealth was approximated by constructing an asset index, not including land, following procedures designed by the Demographic and Health Surveys program (43). We measure household poverty using a multidimensional poverty index that includes indicators of education, health, and living standards (57). Finally, we use a binary measure of food insecurity from household responses of whether they experienced any days in the prior year with food insufficiency (full details in *SI Appendix*, section 1.5).

### Covariate Balance.

Several household- and location-specific characteristics influence agrarian structure. Most notably, agricultural suitability, distance to key infrastructure, population, household size, or gender can explain varying levels in land assets. We selected a full set of baseline covariates from our household survey and available gridded datasets using household geo-locations to extract values (*SI Appendix*, Table S12). For analysis using our covariate selection, we removed households where more than 30% of data are missing, reducing our household survey to n = 994. Missing data for the remaining households are gap-filled by generating multiple imputed datasets (n = 10) with a mix of models for continuous, binary, and ordered data using the *mice* package in R (*SI Appendix*, section 1.3) (62).

We address selection bias on observables by reweighting our covariate sample using entropy balance, implemented in the *MatchThem* package in R, that creates a sample in which treatment is independent of confounders (63, 64). While traditional matching methods are more common than entropy balance, they require a large pool of control candidates not available in our dataset (52% treatment, 48% control). In addition, entropy balance more effectively balances samples than traditional methods, such as propensity score matching, and allows the entire household dataset to be used in our analysis (63).

Prior to adjustment with entropy balance, the selected baseline covariates are already well balanced with standardized mean differences ranging from −0.37 to 0.34 (*SI Appendix*, Table S12). Following reweighting with entropy balance, we reduce standardized mean differences to <0.001 across all covariates (*SI Appendix*, section 1.3).

### Linear and Quantile Models.

We estimate the average treatment effect (ATE) and quantile treatment effect (QTE) of LSLAs on household land assets (log transformed) using weighted cross-sectional models. In the case of linear estimates, we also check the robustness of results with difference-in-differences estimators to account for time-invariant, unobservable confounders (65). For each land asset, we estimate ATEs and QTEs at baseline and post-LSLA (2018). Model specifications include all baseline covariates used in reweighting procedures, entropy balancing weights, and an LSLA site fixed effect. For linear models, we use cluster-robust SEs to construct CIs at the village level (n = 35). SEs are calculated for quantile regressions using the wild bootstrap method with 1,000 replications and clustered at the village level (66). Finally, we pool average and quantile estimates for all gap-filled imputations (n = 10) using Rubin’s rules (*SI Appendix*, section 1.3) (67).

### Land Value.

To examine the associations between LSLAs and land value, we use the National Panel Survey in years 2009 and 2013. For each plot of land, respondents were asked its value “*if it were sold today*” which we use as measures of land value. We normalize self-reported land values by land area, convert to USD, and adjust for inflation between years for an outcome measure of USD per hectare. We then regress our outcome of land value against household distances from our selected LSLAs, land asset size, and survey years (*SI Appendix*, Fig. S1 and Table S6). Additional covariates are included such as proxies of land quality, title, and distances from key infrastructure following prior studies (full details in *SI Appendix*, section 1.4) (68).

### Interaction Effects.

We estimated interaction effects of household baseline characteristics on land asset change using the *interflex* package in R (55). The same covariate control specification was applied as in our linear and quantile models. Our estimates apply weights derived from the entropy balance model and use a kernel estimator to allow for nonlinear interaction effects. Cluster-robust SEs are estimated with 1,000 bootstrap replications at the village level and pooled across gap-filled imputations.

### Mediation Analysis for Well-Being Outcomes.

The effect of LSLAs on household well-being, as mediated through declines in farmland, was investigated using a linear mediation analysis (56). The mediation analysis involves two stages including i) a model of the treatment effect on household farm decreases (binary dependent variable) and ii) an interaction model to differentiate effects of farm decreases on well-being (dependent variable) across treatment and control households. As with the linear and quantile models, we use cross-sectional estimates with entropy balance weights, baseline covariates, LSLA site fixed effects, village-level clustered SEs, and pooled results across all gap-filled imputations. Full details of our mediation analysis are in *SI Appendix*, section 1.5.

### Robustness Checks.

During our 2018 survey, we also collected baseline responses, as is common for impact evaluation studies on natural resource policies where no consistent baseline information is available (69, 70). Some advocate for recall methods over other options in the absence of baseline data but warn against biases, especially over longer periods (60, 71). To help reduce recall bias, our outcome variables focus on salient attributes or rural livelihoods such as land (72). In addition, we used an event calendar at the start of our survey to help households recall the historical period of interest (73). Nevertheless, our survey asks respondents to recall circumstances as early as 2000 that can introduce measurement error into our sample. We checked the robustness of our land asset outcomes by collecting an additional round of surveys in 2019. We found that recall error in land measures is independent of treatment and that our land asset measures are comparable to national surveys (*SI Appendix*, section 1.6).

## Supplementary Material

Appendix 01 (PDF)

## Data Availability

The results, calculated as described in the Methods, are based on the data from the Tanzanian National Bureau of Statistics and the World Bank (https://microdata.worldbank.org/index.php/home) (74–76) and our own household surveys collected in 2018 and 2019 for which we have shared an anonymized version alongside replication code (https://doi.org/10.5281/zenodo.6512230) (77).
