# Supplementary material for: Large-scale land acquisitions exacerbate local farmland inequalities in Tanzania
Source: Proc Natl Acad Sci U S A. 2023 Jul 31;120(32):e2207398120. doi: 10.1073/pnas.2207398120 (PMC10410758; doi:10.1073/pnas.2207398120)
Supplement: Supplementary file 1 — Appendix 01 (PDF) [file pnas.2207398120.sapp.pdf]

PNAS

Supporting Information for

Large-scale land acquisitions exacerbate local farmland inequality in Tanzania

Authors: Jonathan A. Sullivan, Cyrus Samii, Daniel G. Brown, Francis Moyo, Arun Agrawal

Corresponding author:
Jonathan A. Sullivan
jasullivan@arizona.edu

This PDF File Includes

1. Supplemental Text 3

1.1 Quasi-Experimental Design 3

1.2 Data Sources 5

1.3 Empirical Analysis of Land Asset Inequality 6

1.4 Empirical Analysis of Land Value 8

1.5 Mediation Analysis of Well-being Outcomes 10

1.6 Robustness Checks 11

2. Figures 13

Figure S1. Marginal effect plots of the relationship between land asset size, distance from large-scale land acquisitions and land value 13

Figure S2. Pearson correlations of land assets and land rental activity. 14

Figure S4. Comparison across treatment-control households and differing land asset sizes of a) out-migrants' reasons for leaving and b) whether out-migrants send remittances home 16

Figure S5. Comparison of land asset trajectories between treatment and control households. 17

Figure S6. Summary of self-reports of different forms of compensation received 18

Figure S7. Standardized differences for each LSLA site of covariates used in our region growing method to establish control areas. 19

Figure S8. Household covariate balance using the entropy balance algorithm 20

Figure S9. Mean differences in recall error between treatment and control households. 21

Figure S10. Our household survey exhibits comparable landholdings and farm size distributions 22

3. Tables **23**

Table S1. Weighted linear regression results for land assets during pre-acquisitions (baseline), post-acquisition (2018), and difference-in-difference estimates 23

Table S2. Quantile regression model estimates of post-acquisition (2018) household farm size. 25

Table S3. Quantile regression model estimates of post-acquisition (2018) household landholdings. 27

Table S4. Quantile regression model estimates of pre-acquisition (baseline) household farm size. 29

Table S5. Quantile regression model estimates of pre-acquisition (baseline) household landholdings. 31

Table S6. Regression tables for land value estimates. 33

Table S7. Marginal effect of pre-acquisition household income on land asset change 34

Table S8. Marginal treatment effect of pre-acquisition household landholdings on land asset change. 36

Table S9. Marginal effect of pre-acquisition household labor on land asset change). 38

Table S10. Mediation analysis of well-being outcomes. 40

Table S11. List of large-scale land acquisitions compiled from reports and literature. 42

Table S12. Summary of selected villages for our household survey instrument categorized by large-scale land acquisition site and treatment status. 44

Table S13. Geographic covariates used in our region growing algorithm to generate a control zone 45

Table S14. Household sampling intensity was estimated from baseline population estimates and household size. 46

Table S15. Summary of village population at the time of survey collection 47

Table S16. Measures of covariate balance before and after adjustment using entropy balance. 48

Table S17. Summary of each well-being outcome 49

Table S18. Comparison of landholdings and farm size between the National Panel Survey implemented by the Tanzania National Bureau of Statistics. 51

1. Supplemental Text

1.1 Quasi-Experimental Design

1.1.1 Selection of Large-Scale Land Acquisitions

To identify large-scale land acquisitions (LSLAs) in Tanzania for our study, we used available databases including the Land Matrix, existing literature, and government reports at the time of study design in 2015/2016. From these resources, we compiled a set of 25 LSLAs that included geographic location data (Table S11). From these 25 sites, four were selected to ensure that our sample is representative. In previous work we showed that our selected sites (n=4) exhibit no statistical differences from the set of 25 geolocated LSLAs across several gridded datasets including measures of agricultural suitability, population, and distance from transport[1]. In addition, we selected sites to represent a diversity of variables known to differentially affect agricultural change (Table 1).

The universe of LSLAs in Tanzania at the time of study design was greater than the 25 we identified but we were limited to sites with geolocation data required for our analysis. In prior work, we used available data in the Land Matrix to understand how our site selection (n=4) compares to all known LSLA (n=42) in Tanzania[1]. We compare across dimensions of contracted size, prior landowner, prior land use, selected crop, and presence of contract farming. We demonstrated that our selection captures a broad range of LSLA features present in Tanzania. Nevertheless, our selected LSLA sample does not feature land transactions among private owners, those smaller than 1000 ha, or forestry-based investments (see Sullivan et al, 2022 for details).

1.1.2 Treatment Specification & Village Selection

We conducted our analysis at the household level but specified treatment status at the village level. To identify treatment villages, we used geographic definitions of "treatment zones" which were validated during fieldwork in 2017/2018. We define treatment zones for each of our four LSLAs using a five-kilometer buffer surrounding the estimated LSLA boundary. The boundaries of LSLAs are estimated from a combination of geo-locations collected along boundaries during fieldwork in 2017/2018 and the extent of implementation observed from satellite imagery. Previous studies of LSLAs using household datasets also use geographic methods in their identification strategies [2,3]. However, proximity is only an indicator of treatment, at best. To avoid misidentifying treatment villages, and thereby treatment households, we further specified treatment by applying a set of eligibility criteria:

1. At least 50% of the village area falls within the treatment zone
2. Village land must meet one of the following:
    a. fall within the transacted area. For example, village land is being leased to a company for use
    b. now be occupied by the transaction under new tenure
    c. share a boundary with the transaction

Using these eligibility criteria, we refined our definition of treatment beyond a measure of proximity to include villages where customary tenure arrangements are privatized or those that experienced significant changes from LSLA spillovers. Finally, we enumerated all eligible villages within the treatment zones and randomly selected 20 treatment villages to implement our household survey (Table S12).

To select plausible counterfactuals, existing methods often use data on administrative units to match treatment units or randomize interventions. We deviate from this tradition for two reasons. First, administrative boundaries in Tanzania, especially at the village level, are not well defined [4]. Second, with population growth new villages are formed making representations of administrative units at baseline error prone [5]. To avoid these issues, we identify “control zones” with similar socio-ecological characteristics as treatment and apply eligibility criteria to control village selection.

We define control zones for each LSLA site using a region-growing method that identifies a candidate area of the same size as treatment zones. The region growing method minimizes the Mahalanobis distance between treatment and control zones using a set of economic, environmental, and social variables from gridded datasets (Table S13). We also specify that each control zone be outside the treatment zones but within 30-km of the LSLA site and restrict control zones to the same administrative region. The region growing method is initialized by selecting a random pixel, then adding the nearest neighbor pixel based on Mahalanobis distance and is completed when it reaches an equivalent size as the treatment zone. We repeat the region growing procedure 30 times for each LSLA site and select the control zone with the lowest average Mahalanobis distance. Figure S7 demonstrates that our method effectively minimizes the standardized differences between treatment and control Mahalanobis distances. Although individual variables still demonstrate poor balance, this is further addressed in a second-stage, household level matching.

As with treatment villages, we adopted eligibility criteria for control villages:

1. At least 50% of the village area falls within the control area AND
2. The village is not included in the treatment zone

To select control villages, we enumerated all eligible villages and randomly selected 15 where we conducted our household survey (Table S12).

1.1.3 Household Sampling

With the assistance of village chairs and local officials we constructed household rosters for each village. We further subset household rosters to eligible households defined as those residing in villages prior to LSLAs (i.e., pre-acquisition) allowing for baseline characteristics to be collected using recall.

Households were then randomly selected using a stratified random sample based on wealth. To construct proxies for wealth we follow prior studies where households were ranked on a scale from 1 (low) to 4 (high) during focus groups that included village chairs and local officials [6]. We weighted the sampling intensity per LSLA site based on estimates of population[7] and household size[8] and targeted a sample size of either 100 household per site or 2.5% of eligible households (Table S14). In total, we recorded wealth rankings for 9,022 households and

surveyed 1,003 households for our full household survey (Table S12), later reduced to 994 for our analysis.

The household and population count of eligible households (i.e. those residing in villages prior to LSLAs) are comparable between treatment and control villages (Table S15). However, we also observe that treatment villages have populations 55% higher than control villages during fieldwork in 2018 (i.e. post-acquisition) suggesting treatment areas experienced significant in-migration compared to control villages.

1.2 Data Sources

1.2.1 Household Surveys 2018

The principal data for our analysis is a household survey collected March to June 2018 for 1,003 households. A key feature of our survey is its reliance on retrospective questions that require respondents to recall household conditions as far back as 2000. Recall questions are known to be accompanied by measurement error and often can be highly correlated with responses of current conditions, known as "anchoring bias" [9]. Despite these known issues, recall methods remain common within natural resource policy evaluation and several best practices are recommended that we implement here [6,10,11]. Specifically, we use an event calendar to provide a description of a prominent event at baseline to facilitate recollection. For example, for the Hanang Wheat Complex in the north, we asked respondents to think back to President Kikwete's 2005 election to help remember events in 2005. In all cases we also helped participants in the survey recall local events (e.g., local elections, village subdivision) or memorable family matters (e.g., birth of child, house construction) that aid in remembering past household conditions. Finally, our recall question centered on important and easily quantifiable household assets that reduce the complexity of retrospective questions [12].

Our household survey was designed by University of Michigan and International Forestry Resources and Institutions researchers, piloted in Tanzania by the first author in 2017 and subsequently refined. Through our partner institution in Tanzania, Nelson Mandela African Institution of Science and Technology, we recruited eight experienced research assistants (RAs). The first author and field team coordinator trained RAs over the course of two weeks, including review of survey modules, use of Qualtrics tablets where surveys were digitized, use of GPS units, courses on agricultural development policies in Tanzania, and a week of trials in the field. All enumerators were fluent in Kiswahili and English.

The survey included modules on demographics, income, expenditures, land-use, health, fuel use, and food security. In addition, we collected GPS locations of households. Using the Qualtrics server and the *qualtRics* package in R [13], the first author conducted regular quality control checks. In cases of incorrect data, extreme outliers, or missing data RAs were asked to consult field notes or revisit households to address data quality.

The first author obtained a research permit from Tanzania Commission for Science and Technology to conduct fieldwork in Tanzania during 2018 and 2019 (COSTECH Permit No. 2018-443-NA-2018-134). Additionally, an Institutional Review Board exemption was received from the University of Michigan (eResearch ID: HUM00112489).

1.2.2 Household Survey 2019

We collected a second round of household surveys in 2019 for a subsample of 172 households. In the 2019 survey we collected the current conditions of households and the retrospective portion of our survey asked households to recall the year prior (2018). This data was subsequently used to assess recall bias present in our study.

We also conducted semi-structured interviews during 2019 fieldwork. Household selection was driven by our 2018 household survey results and 47 households who experienced land asset gain, loss, and no change were randomly selected in treatment and control areas. Interview questions included reasons behind land asset changes, experienced changes as a result of LSLAs (e.g., employment, infrastructure), changing farming practices, and issues of access to land or farming technology.

1.2.3 National Panel Survey/ Living Standards Measurement Survey

In addition to our household survey, we used the National Panel Survey, also referred to as the Living Standards Measurement Survey, that is a nationally representative survey carried out by the Tanzania National Bureau of Statistics. We accessed the household data for Tanzania via the World Bank's Microdata library for years 2008/09, 2012/13, and 2014/15, herein referred to as years 2009, 2013, and 2015. We drew from years 2009 and 2013 to examine how land values are influenced by land asset sizes and LSLAs using a cross-sectional analysis. In addition, we used years 2009 and 2015 to validate our outcome variables of farm size and landholdings against national distributions of these land assets.

1.3 Empirical Analysis of Land Asset Inequality

For our main analysis of land inequalities and changes in land assets, we used self-reported measures of household farm size and landholdings as our outcome variables fully described in the main manuscript. Below we detail the methods used for our main results including missing data imputation, covariate selection and balance, linear models, and quantile regression models.

1.3.1 Handling of Missing Data

Despite quality control efforts in our household survey, missing data was still present in the final surveys. We filled in missing data with multiple imputations by chained equations using the *mice* package in R [14]. Prior to imputation we removed households where more than 30% of data is missing, reducing our household survey to n=994. To fill in missing data we used classification and regression trees (CART), logistic regression, polynomial regression and ordered logistic regression models for continuous, binary, classified and ordered variables, respectively. For variables that are transformations or groupings of other variables, we used passive imputation methods to maintain linear relationships. To account for uncertainties arising from imputations, we created n=10 imputed datasets and pooled model results in downstream analyses.

1.3.2 Covariates

Baseline covariates were selected from available geographic sources as well as the household survey (Table S16). Geographic covariates include time-independent (e.g., elevation)

and time varying (i.e., landcover) variables. In the case of time-varying covariates, baseline figures were set to the specific start date of LSLA sites. For example, if a given pixel of a time-varying variable fell within the Kilombero Plantation Ltd., acquired in 2008, the value corresponding to 2007 (pre-acquisition) was extracted. Household locations collected using GPS units during fieldwork were used to extract pixel values from raster datasets. For household variable selection, we adopt frameworks of livelihood change and response that consider elements of how institutions, government, or legal frameworks may modify access. In response to changing access to resources, households may draw from several capitals including natural (e.g. land), human (e.g. labor), financial (e.g. income), physical (e.g. assets) and social capitals [15].

As described in the main manuscript, we reweighted household observations based on the selected covariates in our analysis to create a sample that is independent of treatment using the entropy balance algorithm (Figure S8 and Table S16) [16]. Weights determined by the entropy balance algorithm are then input into our linear, quantile and mediation analysis regression estimators.

1.3.3 Estimation Methods

Below we detail the estimation methods used for our main results including linear models and quantile regression models. For all models cluster-robust standard errors are used to construct confidence intervals with clustering at the village level (n=35). In addition, regressions are separately run for each data imputation (n=10) and then pooled using Rubin's rules [17].

Linear models

We use two linear regression models in our examination of land assets. First, we use a weighted linear model to estimate cross-sectional differences between treatment and control baseline land assets. In addition, we use a weighted difference-in-differences specification that is robust to time-invariant unobserved confounders to estimate the average treatment effect of LSLAs. The model specifications for each are provided below.

The model for linear cross-sectional estimates is:

$$Y_i = \beta_0 + \beta_z z_i + x_i' \beta_X + \alpha_i + e_d$$

Where the coefficient $\beta_z$is the average treatment effect of primary interest. x_i is a vector of household-level covariates and β_X is the vector of associated coefficients, while α_i is a fixed-effect for the four selected LSLA sites.

The model for difference-in-differences estimates is:

$$Y_{it} = \beta_0 + \beta_z z_i + \gamma_t t_i + \delta_{dd}(z_i * t_i) + x_i' \beta_X + \alpha_i + e_{dt}$$

Where the coefficient δ_{dd} is the difference-in-differences causal estimate of primary interest. β_z is the coefficient for treatment while γ_t is the time dummy coefficient representing pre- and post-intervention. x_i is a vector of household-level covariates and β_X is the vector of associated coefficients, while α_i is a fixed-effect for the four selected LSLA sites.

Quantile models

We further examine changes in land distribution using a quantile regression model. Here we estimate cross-sectional differences between treatment and control land assets, also referred to as a quantile treatment effect, using weighted quantile regression. The model for quantile τ of the response is:

$$Q_\tau(y_i | [z_i, \alpha_i, x_i]) = \beta_0^{(\tau)} + \beta_z^{(\tau)} z_i + x_i' \beta_X^{(\tau)} + \alpha_i, \qquad i = 1, \ldots, n$$

Where $0 < \tau < 1$ indicates the proportion of the population having y_i values below the τth quantile. The coefficient of interest in our quantile regression model is $\beta_z^{(\tau)}$ representing the quantile treatment effect, which is the effect of the treatment on the value of the τth quantile (e.g., if $\tau = 0.5$, then this corresponds to the effect on the median outcome). x_i is a vector of household-level covariates and $\beta_x^{(\tau)}$ is the vector of associated coefficients, while α_i is a fixed-effect for our four selected LSLA sites.

1.4 Empirical Analysis of Land Value

Land value is an important component of land inequality that, if present in combination with polarization in land asset size, can further widen gaps between the wealthy and poor. Of particular interest in our analysis is whether land values are higher for larger landholders in closer proximity to LSLAs. Here, we examine whether differences in land values are, in part, a function of land asset size, distance from LSLAs, and their interaction. To do so we use the National Panel Survey in a cross-sectional model for years 2009 and 2013, described above, subset to households in the same administrative regions as our selected LSLAs.

1.4.1 Outcomes

The National Panel Survey was previously used to study changing land values in Tanzania by asking respondents, for each plot of land they manage, the value "if it were sold today" [18]. We aggregate land values to the household level for all i) cultivated plots that year and ii) all land managed plots by the household (i.e., includes fallow land). This provides the basis of two outcomes variables of farm size and landholdings used in our land value analysis.

We follow several methods from Wineman & Jayne (2018) in preparing appropriate land value outcome data. First, we adjust for inflation by expressing all land value estimates in 2013 USD. We remove plots that are over 10 kilometers from a household to reduce noise in our LSLA distance estimates that are based on household, not plot, location. This procedure removed 8.9% of actively cultivated plots from our analysis, showing few households manage plots far from their homesteads. Finally, household land values are normalized by land area using either farm size or landholdings. As a result of these steps our outcome variables for our land value analysis are i) household farm value per hectare (USD/ha) and ii) household landholding value per hectare (USD/ha). After sub-setting the National Panel Survey to households within the same administrative region as our selected LSLAs, we had a sample size of n=324 and n=307 for our landholding value and farm value outcomes, respectively.

1.4.2 Covariates

Of interest in our analysis is how land values interact with distance from LSLAs and land asset size. Household GPS locations are provided by the National Panel Survey, although anonymized and randomly offset between 0 to 5 kilometers, that we use to calculate distance from the nearest LSLA border. For land asset size, we create a dummy variable for households below and above the 50th percentile of farm size or landholding. We define the percentile cutoff from *our* household survey so to be comparable to the quantile regression results.

Following Wineman and Jayne (2018), we control for observable confounders available in the National Panel Survey. In particular, we control for agricultural suitability (e.g. slope, soil quality), proximity to infrastructure and markets (e.g. distance from roads, markets), changes in land value over time (e.g. year dummy), and access to land titling.

1.4.3 Estimation Methods

We use a linear regression model to examine the influence of LSLA, land asset size, and interaction effects on land value. Our model for household farm value and land value is:

$$Y_i = \beta_0 + \beta_a a_i + \beta_l l_i + \beta_y y_i + (\beta_{a*l} a * l_i) + (\beta_{a*y} a * y_i) + (\beta_{l*y} l * y_i) + x_i' \beta_X + \alpha_i + e_d$$

Where a_i is the household distance from LSLAs, l_i is whether a household's land assets are below or above the 50th percentile (i.e., a dummy variable), and y_i is a year dummy of the survey. We estimate interaction effects between LSLA distance, land asset size, and survey year. Of particular interest is the interaction between land asset size and LSLA distance indicating whether inequalities exist of land value between small and large landholders in proximity to LSLAs. x_i is a vector of household-level covariates and β_X is the vector of associated coefficients, while α_i is a fixed-effect based on the administrative regions. We use ordinary least squares estimators with cluster-robust standard errors at the region level.

1.4.4 Results

We find no significant effect of land asset size on per hectare land values, though point estimates suggest that smaller farmers experience higher prices (Table S6). The interaction term of distance from LSLAs and land asset percentiles is significant when we consider only cultivated parcels (e.g., farm size), but this effect is small in comparison to insignificant effects of land asset size. Overall, we do not find strong evidence of inequities in land values across gradients of land asset size. Rather, land inequality primarily arises from differences in land assets, specifically farm size as our main analysis demonstrates.

A notable finding in our analysis is that land values decline with greater distances from LSLAs (Figure S1 and Table S6). This effect is stable over time periods included in our analysis and we can only identify associations between LSLAs and land value, not causality without baseline data. This finding, however, furthers our argument that worsening inequality is mediated by household income. Those with higher incomes can expand their farms with new land purchases or rentals despite higher prices. On the other hand, lower income households increasingly are unable to access appreciating land markets. Our land value analysis

demonstrates greater demand or competition for land surrounding LSLAs that, alongside other analyses of household income, suggests a plausible mechanism for how farmland inequality emerges.

1.5 Mediation Analysis of Well-being Outcomes

After finding evidence that LSLA alter farmland distributions, mainly through declines in farmland assets at lower quantiles, we investigate the subsequent impacts on several dimensions of well-being. Below we detail the well-being outcomes selected and the estimation methods to investigate changing land distribution on well-being.

1.5.1 Well-being Outcomes

We further investigate the influence of changing farmland distribution on four well-being outcomes including i) total income, ii) a wealth asset index, iii) a poverty index and iv) a measure of food insecurity. Table S17 summarizes each well-being outcome and the household survey inputs used to construct them.

In our analysis, total income includes all cash and non-cash income from farm and non-farm activities reported by households in Tanzania Shillings (Tsh). Given the skewed nature of income, we normalize reported total income using a log transformation.

We measure household wealth by using an asset index following methodologies from the Demographic Health Surveys (DHS) program. In particular, we compiled a set of indicators for asset ownership and household construction materials that are combined in a principal component analysis. The first principal component explained 23-26% of the variation within the selected indicators and was divided into quintiles representing a wealth index from 1 (poor) to 5 (wealthy).

To estimate poverty, we use the Multi-Dimensional Poverty Index (MPI) developed by the Oxford Poverty and Human Development Initiative[19]. The MPI captures acute deprivations in education, health, living standards and assets that are combined in a weighted index with values ranging from 0 (no deprivations) to 1 (all deprivations).

Finally, food insecurity was reported by households as the number of hunger days experienced in the year prior to the survey. We reduced this measure to a binary variable indicating those that experienced any hunger days in the past year (1) and those with no hunger days (0).

1.5.2 Mediation Analysis of Well-being Outcomes

We use mediation analysis to estimate the degree to which the effect of LSLAs on household well-being passes through farmland decreases - defined as a decline in farm size from baseline to post-acquisition. Our mediation analysis uses a two-stage analysis[20]. The first stage estimates the influence of treatment on the mediator, in this case farmland decreases. The model for the first stage is:

$$\boldsymbol{Stage\ 1}: M_i = \beta_0 + \beta_z z_i + x_i' \beta_X + \alpha_i + e_d$$

Where M_i is the mediator variable representing farmland decrease. Stage two estimates the influence of the mediator on household well-being, the dependent variable, for control and treatment households using an interaction term between treatment status and presence of the mediator. The model for the second stage is:

$$\boldsymbol{Stage\ 2}: Y_i = \beta_0 + \beta_z z_i + \gamma M_i + \delta z_i M_i + x_i'\beta_X + \alpha_i + e_d$$

Where Y_i is a measure of household well-being as a function of the mediator (M_i), treatment (z_i), and their interaction. Using this specification we can estimate the effect of farmland decrease for i) control households using coefficient γ and ii) treatment household by calculating the linear combination of β_z and δ. As with other models in our analysis, specifications for both stages control for household-level covariates and (β_X) and site-level fixed effects (α_i). Since the treatment and mediator are both binary variables, linearity here is innocuous in terms of specification issues, and inclusion of the interaction term makes our analysis more agnostic than classical linear mediation analysis [21]. As with the linear and quantile regressions above, we include entropy balance weights in each stage of the mediation analysis, calculate cluster-robust standard errors at the village level (n=35), and pool results based on each data imputation (n=10) using Rubin's rules.

1.6 Robustness Checks

1.6.1 Recall Bias

During our 2018 survey we collected baseline responses, as is common for impact evaluation studies on natural resource policies where no consistent baseline information is available [10,11]. Some advocate for recall methods over other options in the absence of baseline data but warn against biases, especially over longer periods [9,22]. To help reduce recall bias, our outcome variables focus on salient attributes or rural livelihoods [12]. In addition, we use an event calendar at the start of our survey to help households recall the historical period of interest [6]. Nevertheless, our survey asks respondents to recall circumstances as early as 2000 and measurement error is introduced into our data as is common with these methods [9].

To understand how this measurement error influences our estimates of treatment effects, we check the robustness of our results by evaluating recall bias. To do so, we resurveyed 172 households in 2019 that were randomly selected and asked to recall their circumstances in 2018, the same year as our original survey. Most important for quasi-experimental studies is if recall error is correlated with treatment status, then our estimates are potentially influence by recall bias [23]. Using the same respondents in our 2018 and 2019 surveys, we define recall error as:

$$recall\ error_i = recalled\ land\ asset\ 2018_i - land\ asset\ 2018_i$$

Where the *recalled land asset 2018* are collected in our 2019 survey when respondents are asked to remember their land assets the year prior. The land asset 2018 variable represents that same respondent answer in our 2018 survey of their land assets in that same year. Positive values of

recall error represent respondents who overestimate their prior land assets while negative values are those who underestimate.

We test for mean differences in the recall error across treatment and control households using two-side t-tests and Wilcox tests (Figure S9). We find no significant results in either of our tests for mean differences and conclude that while recall error is present in our sample, it is independent of treatment status.

1.6.2 Consistency of Outcomes with National Data

We compare our land asset distributions to nationally representative data from the National Panel Survey as a robustness check of our data quality. Using the National Panel Survey in years 2009 and 2014 we validated our outcome variables of interest. The difference between median values of the most recent, 2015 National Panel Survey and our household survey in 2018 is 0.01-ha (1.7%) and 0.20-ha (22.0%) for landholdings and farm size, respectively. For the 2008 National Panel Survey compared to our recall household data exhibits a difference of 0.30-ha (18.0%) and 0.33-ha (33.8%) for landholding and farm size, respectively. Larger discrepancies for household recall data are partially explained by two LSLA sites in our sample, Kilombero Sugar Company and Tanganyika Plantation, with pre-acquisition dates in 2000 as compared to the first wave of LSMS in 2009. The LSLA sites with pre-acquisition dates closer to the National Panel Survey 2009, Kilombero Plantation Ltd and Hanang Wheat Complex, differ by 10.9% and 6.4% for landholding and farm size median values, respectively. Moreover, our household dataset and National Panel Survey show consistent distributions across quantiles for both time periods (Figure S10 and Table S18).

2. FIGURES

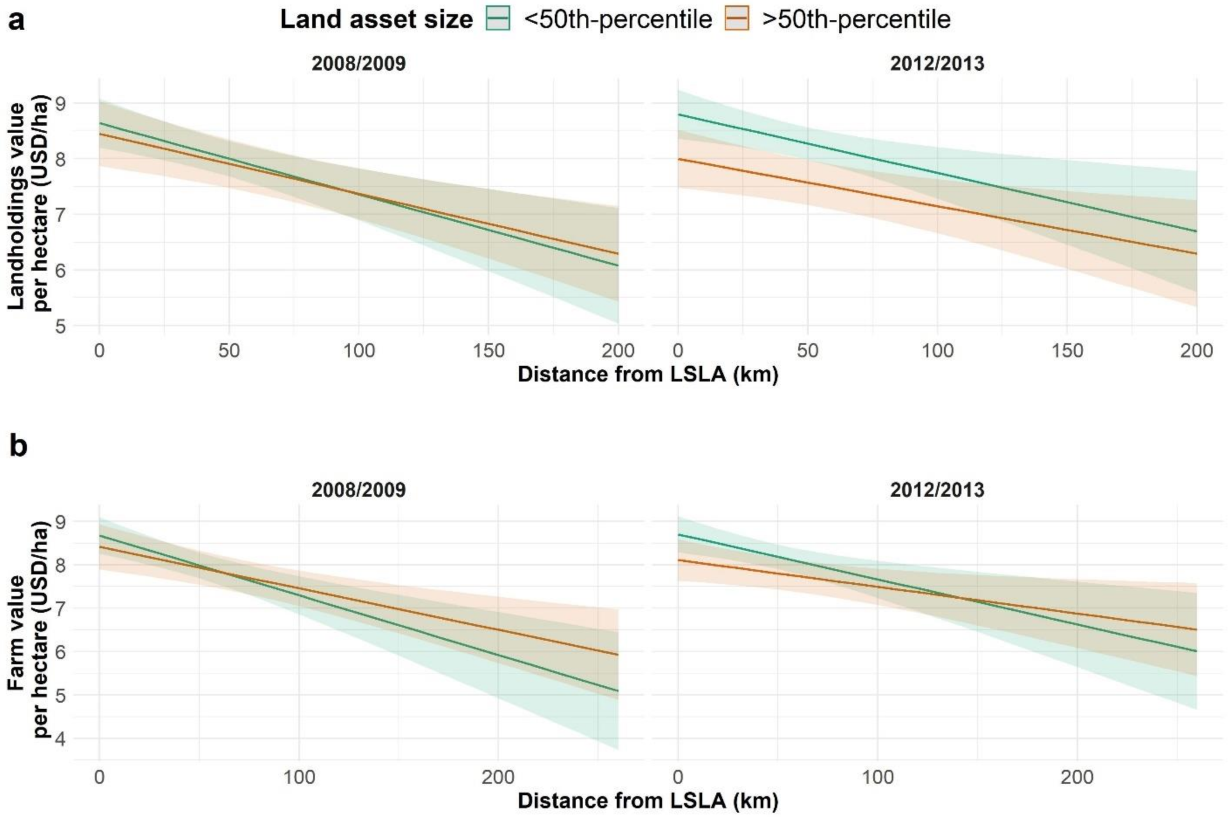

Figure S1. Marginal effect plots of the relationship between land asset size, distance from large-scale land acquisitions (LSLAs) and land value measured as USD/hectare (USD/ha) for a) landholding value and b) farm value. Results show that land values decline as a function of distance from LSLAs, with higher land competition and values closer to LSLAs. Predictions for marginal effect plots are based on coefficients from Table S6 and are created by setting numeric values to the mean and factor terms to their reference level.

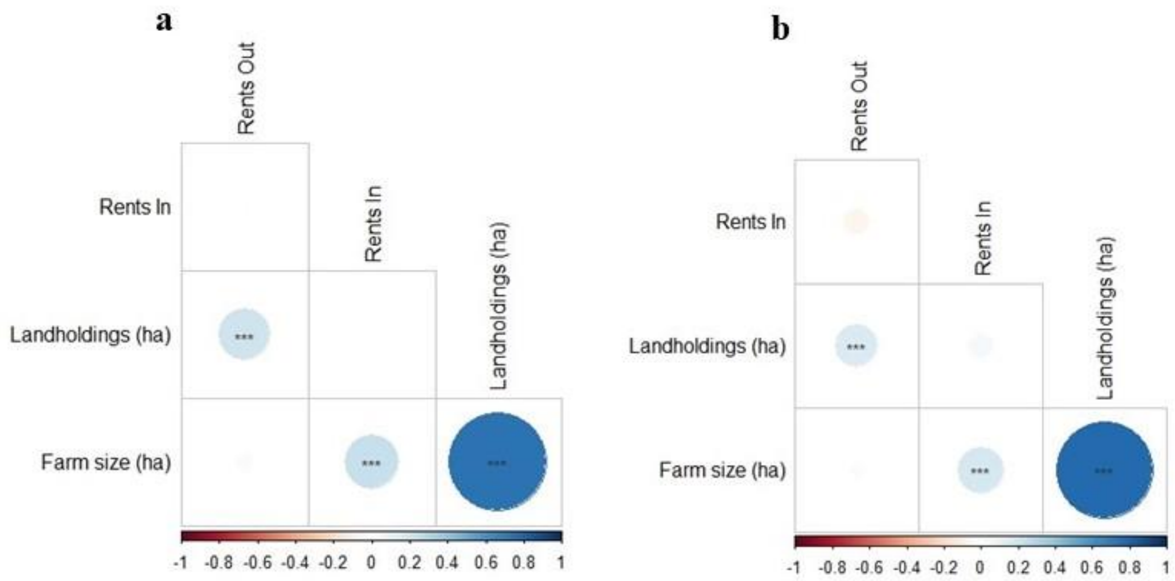

Figure S2. Pearson correlations of land assets and land rental activity for a) treatment and b) control households. The correlation plots show that as farm size increases, land rentals also increase. Similarly, with larger landholdings greater amounts of land rental activity is present. These patterns hold true for both control and treatment households. Significance levels denoted by *** $p < 0.001$; ** $p < 0.01$; * $p < 0.05$.

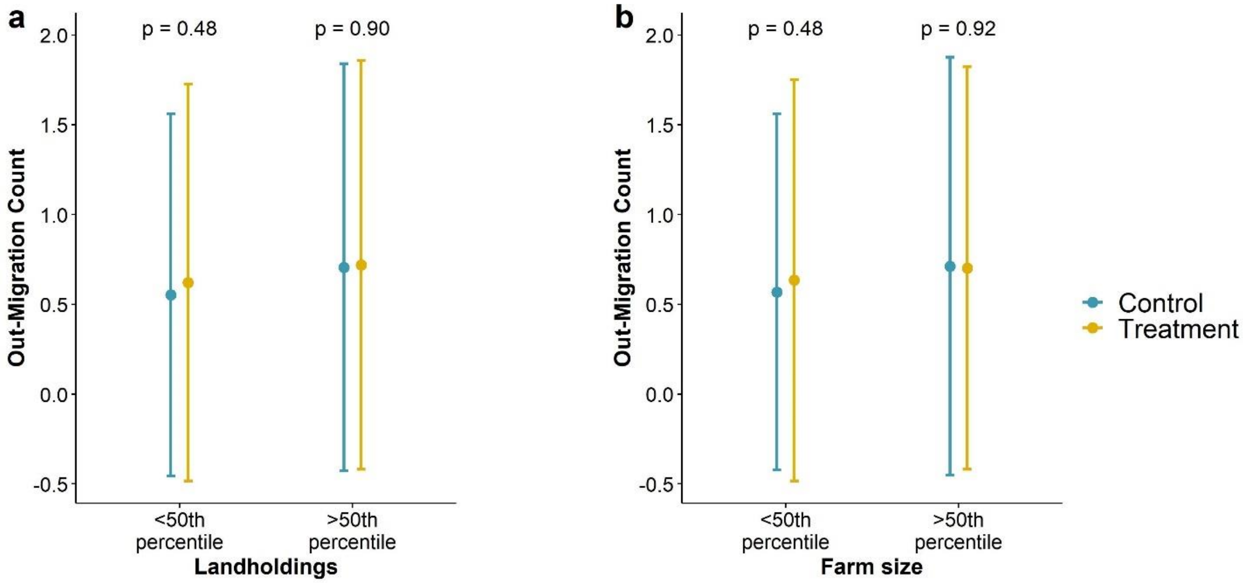

Figure S3. Comparison of out-migration rates between treatment and control households and across groupings of land asset size for a) landholdings and b) farm size. In our survey respondents report the number of household members that have out-migrated since the start of LSLAs. Based on p-values using two-sided t-tests between treatment and control households, we find no significant difference between out-migration. This finding holds for households with either small (<50th-percentile) or large (>50th-percentile) land assets. Error bars represent standard deviation from the mean.

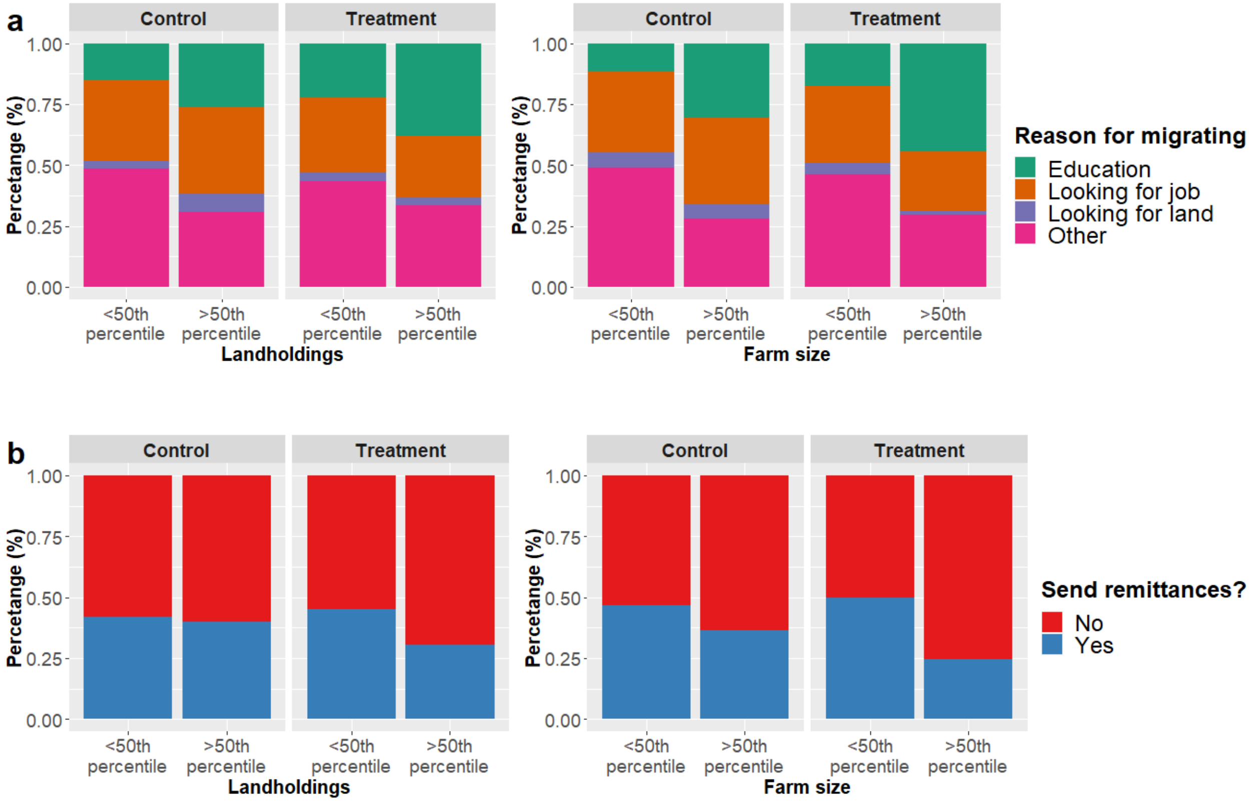

Figure S4. Comparison across treatment-control households and differing land asset sizes of a) out-migrants' reasons for leaving and b) whether out-migrants send remittances home. Based on data from our household survey, higher rates of out-migrants from households above the 50th-percentile in land assets seek education, with a pronounced effect in treatment households. Though fewer of these out-migrants send remittances home.

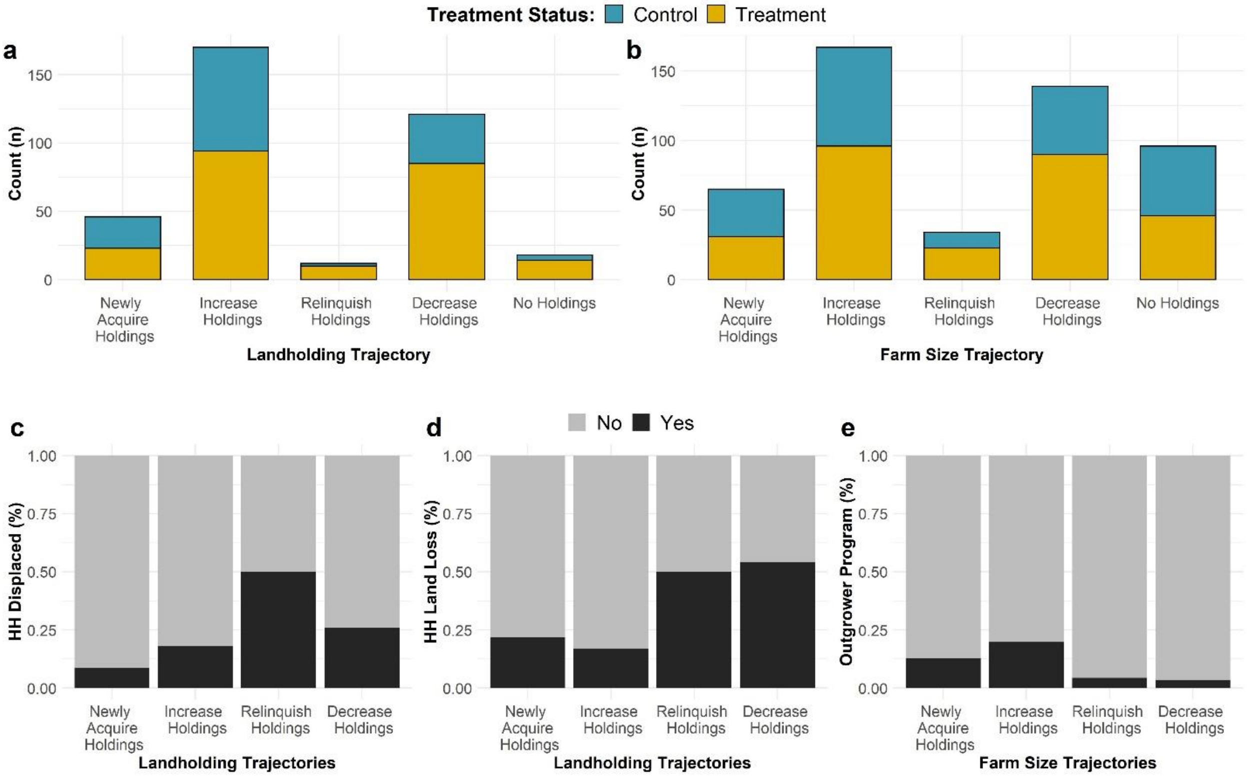

Figure S5. Comparison of land asset trajectories between treatment and control households as well as LSLA mechanisms of displacement, land loss, and contract farming. Land asset trajectories include those who newly acquire holdings, increase holdings, relinquish holdings, or decrease holdings and explain proportions of households who **a)** change their landholdings and **b)** farm size. In addition, LSLAs show varying effects of different land assets trajectories conditional on whether households were **c)** displaced by LSLAs, **d)** lost land to LSLAs and **e)** participated in contract farming programs hosted by LSLAs.

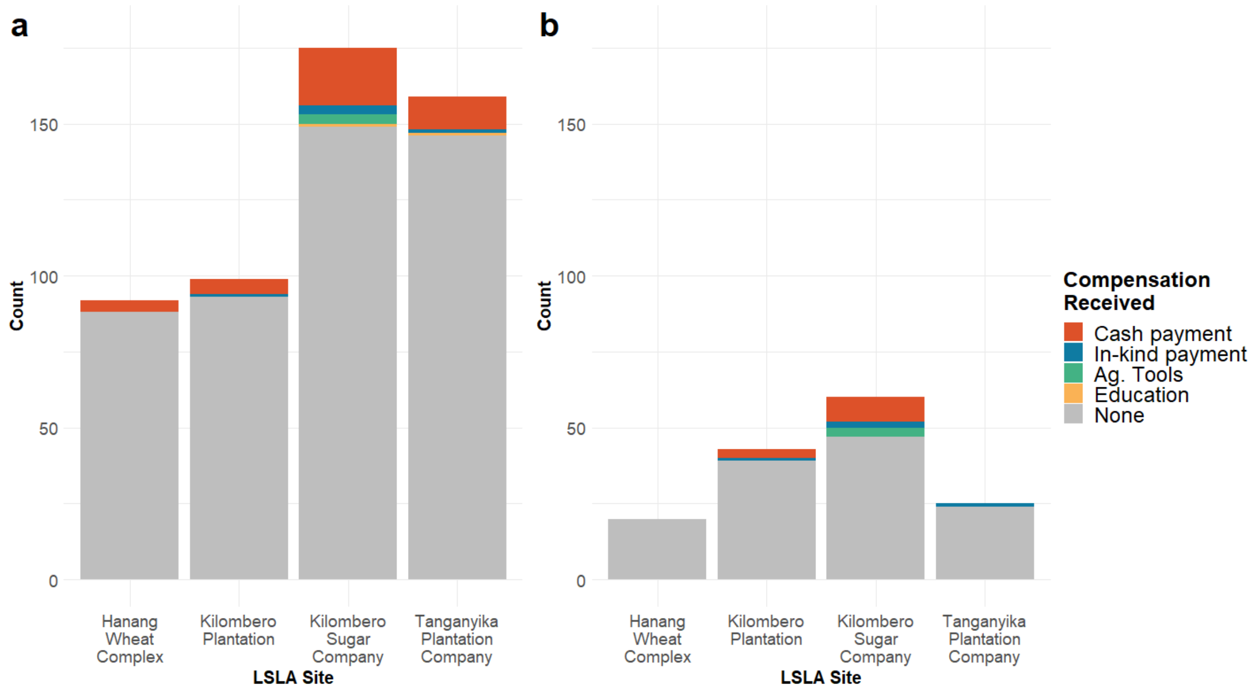

Figure S6. Summary of self-reports of different forms of compensation received by **a)** all the households in the treatment area and **b)** households that reported being displaced or losing farmland due to LSLAs.

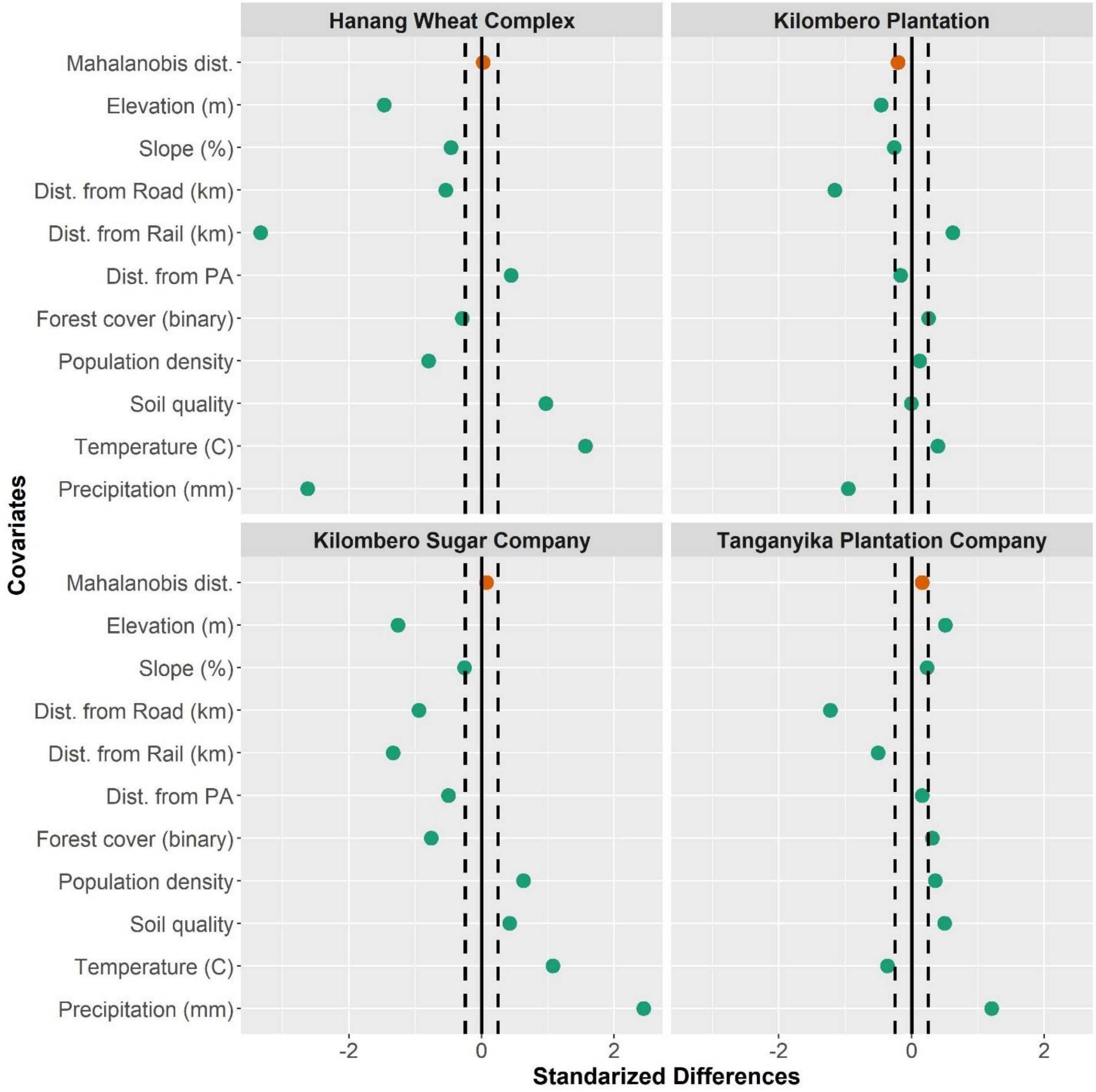

Figure S7. Standardized differences for each LSLA site of covariates used in our region growing method to establish control areas from which control villages are enumerated and then randomly selected. The region growing method minimizes the Mahalanobis distance of all covariates which is effectively balanced. To estimate standardized differences displayed, covariate values are randomly sampled (n=1,000) from gridded datasets in both treatment and control areas. Balance thresholds of 0.25 and -0.25 suggested by Stuart (2010) are shown as dashed lines [24].

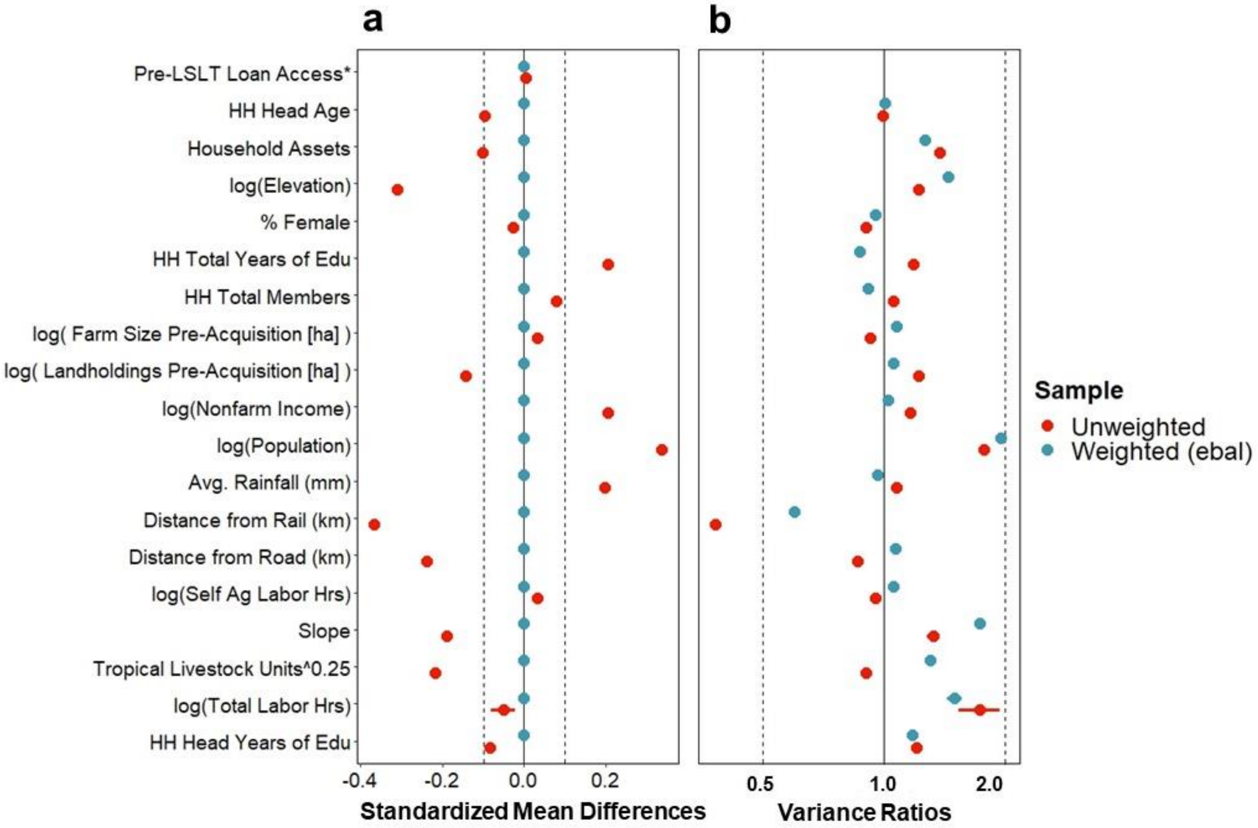

Figure S8. Household covariate balance before and after adjustment using the entropy balance algorithm for a) standardized mean differences and **b)** variance ratios. Reweighted household observations are used in linear and quantile regression specifications to create a sample independent of treatment. Calculations for the standardized mean differences use pooled standard deviations most appropriate for computing the Average Treatment Effect (ATE). Ranges represent standard errors from multiple imputations of the household survey dataset (n=10). HH = household.

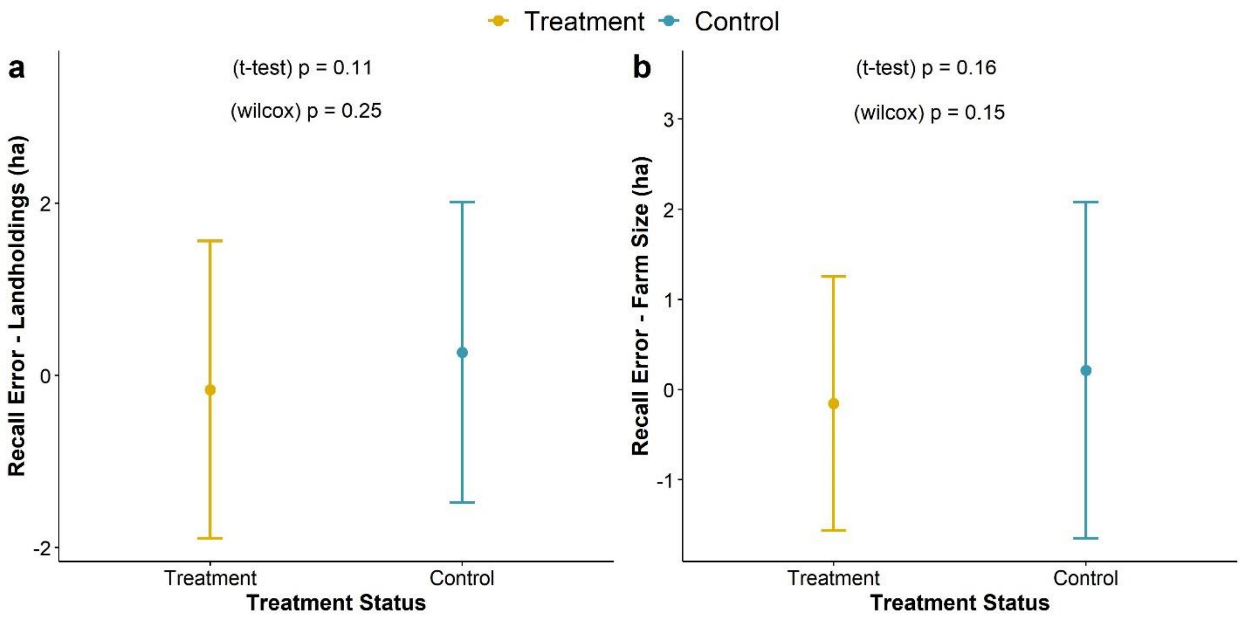

Figure S9. Mean differences in recall error between treatment and control households for a) landholdings and b) farm size. Recall error was measured as the difference between recalled land assets in 2018 (collected in 2019) and reported land assets in 2018. We use two-sided t-tests and Wilcox tests to evaluate mean differences across treatment and control households and find no significant results. Error bars represent standard deviation from the mean.

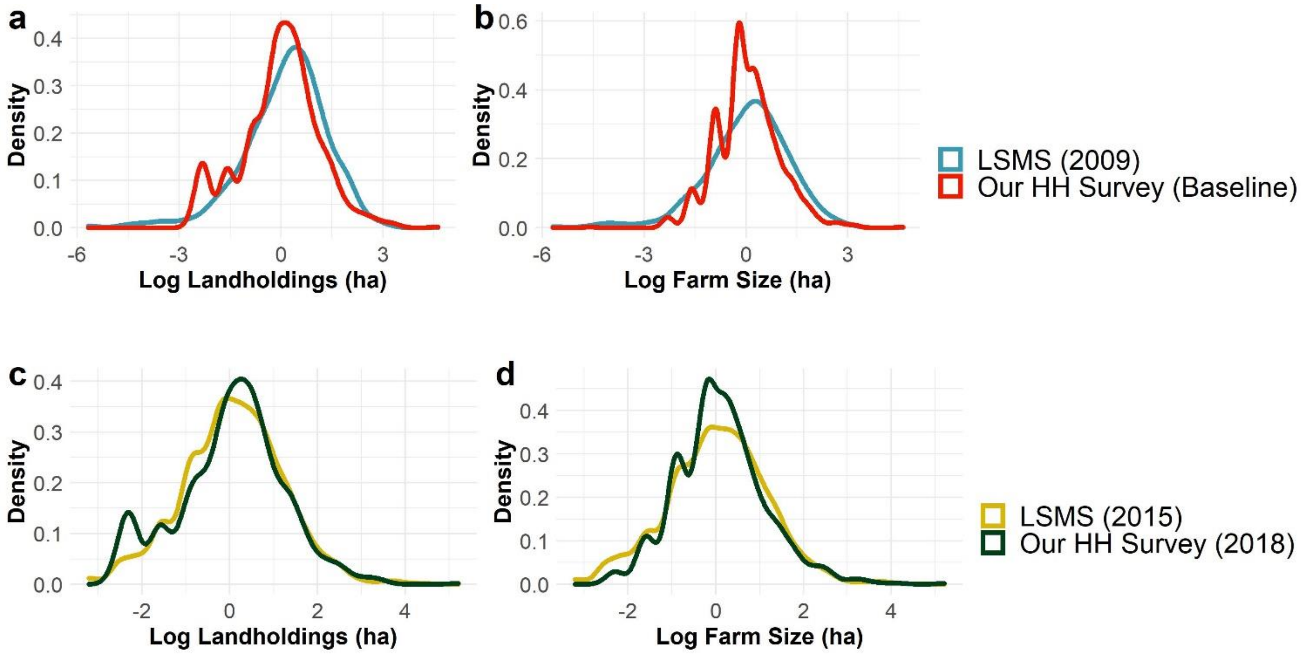

Figure S10. Our household survey exhibits comparable landholdings and farm size distributions to a, b) the National Panel Survey for 2009, and **c, d)** the National Panel Survey for 2015.

3. TABLES

Table S1. Weighted linear regression results for land assets during pre-acquisitions (baseline), post-acquisition (2018), and difference-in-difference estimates. Bolded values are reported in Figure 2 of the main manuscript. Standard errors reported in parentheses. Significance levels denoted by *** p < 0.001; ** p < 0.01; * p < 0.05. Data transformations denoted by † = log transformed; ‡ = double-square root transformed.

	**Landholdings**			**Farm size**		
	**Placebo**	**2018**	**Diff-in-Diff**	**Placebo**	**2018**	**Diff-in-Diff**
**Treatment Status ($\boldsymbol{\beta_z}$)**	**-0.02 (0.10)**	**-0.34 ** (0.12)**	-0.07 (0.11)	**-0.08 (0.21)**	**-0.44 *** (0.10)**	-0.24 (0.20)
Time dummy (β_{post})			0.37 *** (0.07)			0.28 ** (0.10)
**Treatment * Time ($\boldsymbol{\beta_{dd}}$)**			**-0.24 * (0.10)**			**-0.08 (0.16)**
Elevation (m) †	-0.46 (0.36)	0.14 (0.26)	-0.26 (0.32)	0.57 (0.34)	-0.70 (0.36)	0.06 (0.34)
Slope (degrees)	-0.01 (0.01)	-0.00 (0.01)	-0.01 (0.01)	0.00 (0.01)	0.00 (0.02)	0.00 (0.01)
Distance from road (km)	0.03 (0.03)	0.06 * (0.02)	0.05 * (0.03)	-0.05 (0.04)	0.15 *** (0.03)	0.04 (0.03)
Distance from rail (km)	0.00 (0.00)	-0.00 (0.01)	-0.00 (0.01)	-0.01 (0.01)	-0.01 (0.01)	-0.01 * (0.00)
Population †	-0.04 (0.07)	-0.10 (0.06)	-0.08 (0.07)	-0.06 (0.07)	-0.01 (0.08)	-0.05 (0.07)
Mean precipitation	-0.01 (0.01)	0.02 * (0.01)	0.01 (0.01)	0.02 (0.02)	0.04 *** (0.01)	0.03 * (0.01)
Baseline farm size (ha)	0.55 *** (0.06)	0.07 (0.04)	0.43 *** (0.04)		0.45 *** (0.08)	
Baseline landholdings (ha)		0.42 *** (0.06)		0.67 *** (0.05)	-0.01 (0.08)	0.48 *** (0.04)
Household size	0.04 (0.04)	0.09 *** (0.02)	0.07 * (0.03)	0.01 (0.03)	0.06 * (0.03)	0.04 (0.03)
Percent household female	0.03 (0.15)	0.22 (0.14)	0.13 (0.12)	-0.17 (0.19)	-0.04 (0.20)	-0.14 (0.19)
Household head education (years)	0.01 (0.02)	0.04 * (0.02)	0.02 (0.01)	0.03 (0.02)	0.09 *** (0.02)	0.06 *** (0.02)
Household total years of education (years)	-0.01 (0.04)	-0.01 (0.03)	-0.02 (0.03)	0.05 (0.04)	0.01 (0.03)	0.04 (0.04)
Head age (years)†	0.41 * (0.18)	-0.13 (0.14)	0.22 (0.13)	-0.04 (0.13)	-0.44 * (0.18)	-0.25 * (0.12)
Nonfarm income (Tsh)†	-0.01 * (0.01)	-0.00 (0.01)	-0.01 ** (0.00)	0.00 (0.01)	-0.00 (0.01)	-0.00 (0.01)
	0.19	0.08	0.17	-0.19	0.06	-0.11

	Landholdings			Farm size		
	Placebo	2018	Diff-in-Diff	Placebo	2018	Diff-in-Diff
Tropical livestock units‡	(0.11)	(0.07)	(0.09)	(0.17)	(0.08)	(0.15)
Household loan (binary)	0.22	0.30 *	0.30 *	0.10	0.19	0.17
	(0.15)	(0.14)	(0.12)	(0.23)	(0.16)	(0.18)
Total labor (hours)†	-0.05	0.18	0.06	-0.85 ***	0.56 *	-0.34
	(0.16)	(0.14)	(0.13)	(0.21)	(0.24)	(0.17)
Self ag labor (hours)†	-0.00	-0.06	-0.03	0.99 ***	0.09	0.77 ***
	(0.12)	(0.10)	(0.10)	(0.15)	(0.19)	(0.13)
Household assets (count)	-0.14 ***	-0.06	-0.13 ***	-0.07 *	-0.05	-0.08 *
	(0.03)	(0.03)	(0.03)	(0.03)	(0.05)	(0.03)
Fixed Effect - KPL	-0.15	-1.17	-0.69	-0.94	-4.24 ***	-2.80 **
	(1.16)	(0.98)	(1.13)	(1.38)	(0.86)	(0.98)
Fixed Effect - KSC	-0.20	-0.99	-0.64	-0.83	-3.52 ***	-2.36 **
	(0.93)	(0.77)	(0.89)	(1.11)	(0.73)	(0.83)
Fixed Effect - TPC	-0.36	-0.55	-0.53	-0.46	-1.89 ***	-1.28 **
	(0.48)	(0.45)	(0.47)	(0.55)	(0.54)	(0.49)
Intercept	3.52	-1.20	1.71	-3.89	3.78	-1.07
	(2.99)	(2.28)	(2.60)	(2.96)	(3.05)	(2.98)

Table S2. Quantile regression model estimates of post-acquisition (2018) household farm size. Bolded values are reported in Figure 2 of the main manuscript. Standard errors reported in parentheses. († = log transformed, ‡ = double-square root transformed).

term	tau (τ)									
	**0.05**	**0.15**	**0.25**	**0.35**	**0.45**	**0.55**	**0.65**	**0.75**	**0.85**	**0.95**
(Intercept)	-0.96 (11.6)	0.35 (3.37)	1.04 (1.66)	0.00 (0.95)	0.00 (0.84)	0.00 (1.95)	2.99 (2.97)	2.41 (3.04)	2.75 (3.49)	9.84 (1838.25)
**Treatment Status (β_z)**	**-1.24 (0.45)**	**-0.61 (0.24)**	**-0.35 (0.21)**	**0.00 (0.16)**	**0.00 (0.1)**	**0.00 (0.13)**	**-0.16 (0.14)**	**-0.15 (0.13)**	**-0.07 (0.14)**	**0.03 (5.01)**
Fixed Effect - KPL	-3.69 (8.08)	-3.60 (1.53)	-2.49 (1.44)	0.00 (1.23)	0.00 (0.89)	0.00 (1.32)	-2.51 (1.14)	-1.80 (1.21)	-1.63 (1.24)	-1.11 (561.83)
Fixed Effect - KSC	-2.80 (7.68)	-3.03 (1.3)	-2.06 (1.17)	0.00 (1.01)	0.00 (0.74)	0.00 (1.09)	-2.09 (0.92)	-1.53 (0.97)	-1.43 (1)	-0.97 (546.31)
Fixed Effect - TPC	1.22 (6.67)	-0.80 (0.74)	-0.71 (0.5)	0.00 (0.42)	0.00 (0.35)	0.00 (0.59)	-1.35 (0.59)	-1.43 (0.61)	-1.72 (0.66)	-3.19 (249.61)
Elevation (m)†	-1.07 (1.13)	-0.11 (0.36)	-0.15 (0.18)	0.00 (0.11)	0.00 (0.1)	0.00 (0.24)	-0.47 (0.36)	-0.32 (0.37)	-0.26 (0.41)	-0.71 (259.54)
Slope (degrees)	0.00 (0.05)	0.01 (0.02)	0.01 (0.01)	0.00 (0.01)	0.00 (0.01)	0.00 (0.01)	0.00 (0.01)	0.00 (0.01)	-0.01 (0.01)	-0.02 (0.76)
Distance from road (km)	0.13 (0.1)	0.04 (0.04)	0.03 (0.02)	0.00 (0.02)	0.00 (0.02)	0.00 (0.04)	0.11 (0.04)	0.14 (0.04)	0.12 (0.05)	0.05 (1.18)
Distance from rail (km)	0.03 (0.05)	-0.01 (0.01)	-0.01 (0.01)	0.00 (0.01)	0.00 (0)	0.00 (0.01)	-0.01 (0.01)	0.00 (0.01)	-0.01 (0.01)	-0.01 (0.59)
Population†	0.00 (0.21)	-0.04 (0.07)	-0.02 (0.04)	0.00 (0.02)	0.00 (0.02)	0.00 (0.04)	0.03 (0.05)	0.08 (0.06)	0.06 (0.09)	0.21 (3.01)
Mean precipitation (mm)	0.07 (0.04)	0.04 (0.02)	0.02 (0.01)	0.00 (0.01)	0.00 (0.01)	0.00 (0.01)	0.02 (0.01)	0.02 (0.01)	0.01 (0.01)	-0.02 (1.26)
Landholdings (ha)†	0.02 (0.1)	-0.02 (0.05)	-0.02 (0.03)	0.00 (0.03)	0.00 (0.03)	0.00 (0.06)	0.02 (0.09)	0.10 (0.08)	0.07 (0.08)	0.00 (0.67)
Farm size (ha)†	0.47 (0.12)	0.79 (0.08)	0.86 (0.08)	1.00 (0.1)	1.00 (0.11)	1.00 (0.19)	0.61 (0.21)	0.30 (0.1)	0.22 (0.08)	0.14 (0.62)
Household size	0.05 (0.05)	0.02 (0.03)	0.04 (0.02)	0.00 (0.02)	0.00 (0.02)	0.00 (0.04)	0.07 (0.03)	0.09 (0.03)	0.08 (0.04)	0.07 (0.33)

Percent household female	-0.11<br>(0.36)	-0.23<br>(0.21)	-0.08<br>(0.13)	0.00<br>(0.08)	0.00<br>(0.06)	0.00<br>(0.09)	0.11<br>(0.16)	0.09<br>(0.2)	0.16<br>(0.23)	0.06<br>(2.66)
Household head education (years)	0.06<br>(0.03)	0.02<br>(0.02)	0.01<br>(0.01)	0.00<br>(0.01)	0.00<br>(0.01)	0.00<br>(0.02)	0.06<br>(0.02)	0.08<br>(0.02)	0.10<br>(0.02)	0.09<br>(0.19)
Household total years of education (years)	0.06<br>(0.07)	0.03<br>(0.04)	-0.01<br>(0.02)	0.00<br>(0.01)	0.00<br>(0.01)	0.00<br>(0.02)	-0.03<br>(0.03)	-0.02<br>(0.04)	0.02<br>(0.04)	-0.01<br>(0.48)
Head age (years)†	-0.69<br>(0.31)	-0.48<br>(0.17)	-0.30<br>(0.14)	0.00<br>(0.15)	0.00<br>(0.12)	0.00<br>(0.17)	-0.25<br>(0.17)	-0.19<br>(0.19)	-0.11<br>(0.24)	-0.10<br>(2.22)
Nonfarm income (Tsh)†	-0.03<br>(0.02)	-0.01<br>(0.01)	0.00<br>(0)	0.00<br>(0)	0.00<br>(0)	0.00<br>(0.01)	-0.01<br>(0.01)	-0.02<br>(0.01)	-0.02<br>(0.01)	-0.01<br>(0.09)
Tropical livestock units‡	0.05<br>(0.34)	0.02<br>(0.08)	-0.01<br>(0.05)	0.00<br>(0.02)	0.00<br>(0.02)	0.00<br>(0.03)	-0.02<br>(0.05)	0.02<br>(0.07)	0.00<br>(0.08)	-0.08<br>(7.41)
Household loan (binary)	0.73<br>(146.53)	0.23<br>(17.66)	0.10<br>(19.55)	0.00<br>(11.82)	0.00<br>(0.1)	0.00<br>(0.16)	0.07<br>(9.69)	0.07<br>(24.36)	0.05<br>(251.35)	-0.17<br>(2016.61)
Total labor (hours)†	0.35<br>(0.32)	0.15<br>(0.15)	0.09<br>(0.12)	0.00<br>(0.1)	0.00<br>(0.08)	0.00<br>(0.14)	-0.07<br>(0.23)	-0.24<br>(0.22)	-0.23<br>(0.18)	0.09<br>(1.95)
Self ag labor (hours)†	1.34<br>(0.59)	0.29<br>(0.5)	0.12<br>(0.2)	0.00<br>(0.11)	0.00<br>(0.09)	0.00<br>(0.2)	0.45<br>(0.27)	0.46<br>(0.28)	0.38<br>(0.25)	0.19<br>(2.53)
Household assets (count)	-0.02<br>(0.08)	-0.02<br>(0.03)	-0.02<br>(0.02)	0.00<br>(0.02)	0.00<br>(0.01)	0.00<br>(0.02)	-0.04<br>(0.03)	-0.07<br>(0.04)	-0.09<br>(0.05)	-0.11<br>(0.4)

Table S3. Quantile regression model estimates of post-acquisition (2018) household landholdings. Bolded values are reported in Figure 2 of the main manuscript. Standard errors reported in parentheses. († = log transformed, ‡ = double-square root transformed).

term	tau (τ)									
	**0.05**	**0.15**	**0.25**	**0.35**	**0.45**	**0.55**	**0.65**	**0.75**	**0.85**	**0.95**
(Intercept)	6.16<br>(10.19)	0.17<br>(1.84)	-0.01<br>(1.19)	-0.01<br>(0.46)	0.00<br>(0.25)	0.00<br>(0.63)	1.42<br>(1.52)	3.31<br>(2.3)	1.36<br>(4.74)	-3.05<br>(1021.56)
**Treatment Status (β_z)**	**-0.91<br>(0.53)**	**-0.34<br>(0.22)**	**-0.01<br>(0.14)**	**0.00<br>(0.05)**	**0.00<br>(0.02)**	**0.00<br>(0.06)**	**-0.15<br>(0.11)**	**-0.20<br>(0.14)**	**-0.23<br>(0.17)**	**0.16<br>(1.36)**
Fixed Effect - KPL	-2.44<br>(6.38)	-1.56<br>(1.23)	-0.02<br>(0.74)	0.00<br>(0.26)	-0.01<br>(0.16)	-0.01<br>(0.5)	-0.77<br>(0.8)	-0.64<br>(1.16)	-0.59<br>(2)	1.99<br>(313.71)
Fixed Effect - KSC	-2.31<br>(4.4)	-1.46<br>(1)	-0.02<br>(0.63)	0.00<br>(0.23)	0.00<br>(0.14)	-0.01<br>(0.42)	-0.75<br>(0.64)	-0.57<br>(0.91)	-0.47<br>(1.77)	1.95<br>(304.28)
Fixed Effect - TPC	-1.03<br>(3.66)	-0.39<br>(0.47)	-0.01<br>(0.24)	0.00<br>(0.08)	0.00<br>(0.06)	0.00<br>(0.23)	-0.57<br>(0.38)	-0.93<br>(0.46)	-1.32<br>(0.81)	-0.38<br>(126.45)
Elevation (m)†	-0.69<br>(1.22)	0.02<br>(0.24)	0.00<br>(0.17)	0.00<br>(0.06)	0.00<br>(0.02)	0.00<br>(0.07)	-0.22<br>(0.2)	-0.36<br>(0.33)	0.08<br>(0.73)	0.98<br>(144.58)
Slope (degrees)	0.03<br>(0.04)	0.01<br>(0.01)	0.00<br>(0.01)	0.00<br>(0)	0.00<br>(0)	0.00<br>(0)	0.00<br>(0.01)	0.00<br>(0.01)	-0.02<br>(0.02)	-0.05<br>(0.46)
Distance from road (km)	0.04<br>(0.09)	0.02<br>(0.03)	0.00<br>(0.01)	0.00<br>(0)	0.00<br>(0)	0.00<br>(0.01)	0.04<br>(0.02)	0.06<br>(0.02)	0.11<br>(0.04)	0.10<br>(0.36)
Distance from rail (km)	-0.01<br>(0.03)	-0.01<br>(0.01)	0.00<br>(0)	0.00<br>(0)	0.00<br>(0)	0.00<br>(0)	0.00<br>(0)	0.00<br>(0.01)	-0.01<br>(0.01)	0.00<br>(0.19)
Population†	-0.03<br>(0.17)	-0.05<br>(0.05)	0.00<br>(0.03)	0.00<br>(0.02)	0.00<br>(0.02)	0.00<br>(0.03)	-0.02<br>(0.03)	-0.02<br>(0.04)	0.04<br>(0.1)	0.12<br>(1.22)
Mean precipitation (mm)	0.01<br>(0.03)	0.02<br>(0.01)	0.00<br>(0.01)	0.00<br>(0)	0.00<br>(0)	0.00<br>(0)	0.01<br>(0.01)	0.00<br>(0.01)	0.00<br>(0.02)	-0.01<br>(0.75)
Landholdings (ha)†	0.76<br>(0.14)	0.87<br>(0.07)	1.00<br>(0.06)	1.00<br>(0.04)	1.00<br>(0.04)	1.00<br>(0.13)	0.60<br>(0.14)	0.47<br>(0.1)	0.23<br>(0.11)	0.12<br>(0.52)
Farm size (ha)†	0.05<br>(0.12)	-0.01<br>(0.05)	0.00<br>(0.02)	0.00<br>(0.01)	0.00<br>(0.01)	0.00<br>(0.02)	0.05<br>(0.04)	0.05<br>(0.05)	0.11<br>(0.05)	0.13<br>(0.32)
Household size	0.10<br>(0.06)	0.05<br>(0.01)	0.00<br>(0.02)	0.00<br>(0.01)	0.00<br>(0.01)	0.00<br>(0.02)	0.07<br>(0.03)	0.05<br>(0.03)	0.09<br>(0.04)	0.11<br>(0.19)

Percent household female	-0.06<br>(0.44)	-0.06<br>(0.09)	0.00<br>(0.05)	0.00<br>(0.02)	0.00<br>(0.02)	0.00<br>(0.06)	0.18<br>(0.09)	0.14<br>(0.16)	0.29<br>(0.23)	0.25<br>(1.58)
Household head education (years)	0.01<br>(0.03)	0.01<br>(0.01)	0.00<br>(0.01)	0.00<br>(0)	0.00<br>(0)	0.00<br>(0.01)	0.02<br>(0.01)	0.03<br>(0.02)	0.05<br>(0.03)	0.02<br>(0.09)
Household total years of education (years)	-0.04<br>(0.06)	-0.02<br>(0.02)	0.00<br>(0.01)	0.00<br>(0)	0.00<br>(0)	0.00<br>(0.01)	-0.03<br>(0.03)	0.02<br>(0.03)	0.02<br>(0.05)	0.02<br>(0.17)
Head age (years)†	-0.45<br>(0.27)	-0.27<br>(0.11)	0.00<br>(0.08)	0.00<br>(0.02)	0.00<br>(0.02)	0.00<br>(0.06)	-0.06<br>(0.13)	-0.09<br>(0.15)	-0.12<br>(0.22)	-0.27<br>(0.87)
Nonfarm income (Tsh)†	0.00<br>(0.01)	0.00<br>(0)	0.00<br>(0)	0.00<br>(0)	0.00<br>(0)	0.00<br>(0)	0.00<br>(0.01)	-0.01<br>(0.01)	-0.02<br>(0.01)	-0.02<br>(0.04)
Tropical livestock units‡	0.06<br>(0.53)	0.05<br>(0.03)	0.00<br>(0.02)	0.00<br>(0.01)	0.00<br>(0.01)	0.00<br>(0.02)	0.03<br>(0.04)	0.01<br>(0.07)	0.02<br>(0.09)	-0.15<br>(4.33)
Household loan (binary)	0.28<br>(97.36)	0.10<br>(40.21)	0.00<br>(3.4)	0.00<br>(0.06)	0.00<br>(0.09)	0.00<br>(0.15)	0.15<br>(6.89)	0.23<br>(44.33)	0.10<br>(226.14)	-0.10<br>(2011.21)
Total labor (hours)†	-0.03<br>(0.24)	-0.02<br>(0.07)	0.00<br>(0.03)	0.00<br>(0.01)	0.00<br>(0.01)	0.00<br>(0.03)	0.02<br>(0.1)	-0.10<br>(0.15)	-0.13<br>(0.2)	-0.03<br>(0.84)
Self ag labor (hours)†	-0.04<br>(0.47)	0.09<br>(0.12)	0.00<br>(0.07)	0.00<br>(0.02)	0.00<br>(0.02)	0.00<br>(0.04)	0.06<br>(0.12)	0.21<br>(0.19)	0.17<br>(0.24)	-0.04<br>(0.95)
Household assets (count)	-0.05<br>(0.08)	-0.02<br>(0.03)	0.00<br>(0.01)	0.00<br>(0)	0.00<br>(0)	0.00<br>(0.01)	-0.02<br>(0.02)	-0.04<br>(0.03)	-0.08<br>(0.04)	-0.14<br>(0.23)

Table S4. Quantile regression model estimates of pre-acquisition (baseline) household farm size. Bolded values are reported in Figure 2 of the main manuscript. Standard errors reported in parentheses. († = log transformed, ‡ = double-square root transformed).

term	tau (τ)									
	**0.05**	**0.15**	**0.25**	**0.35**	**0.45**	**0.55**	**0.65**	**0.75**	**0.85**	**0.95**
(Intercept)	-2.18<br>(16.59)	-4.86<br>(5.92)	-1.39<br>(3.86)	-0.38<br>(2.5)	-0.10<br>(1.29)	0.32<br>(0.68)	0.54<br>(0.51)	0.56<br>(0.71)	1.26<br>(2)	-4.83<br>(4.77)
**Treatment Status ($\boldsymbol{\beta_z}$)**	**-0.43<br>(0.72)**	**-0.30<br>(0.38)**	**-0.07<br>(0.18)**	**-0.03<br>(0.08)**	**0.03<br>(0.05)**	**0.02<br>(0.03)**	**0.01<br>(0.02)**	**0.01<br>(0.04)**	**0.08<br>(0.12)**	**0.25<br>(0.2)**
Fixed Effect - KPL	-2.53<br>(6.19)	-2.21<br>(2.7)	-0.62<br>(1.49)	-0.31<br>(0.82)	-0.23<br>(0.43)	-0.23<br>(0.23)	-0.25<br>(0.19)	-0.22<br>(0.29)	-0.24<br>(0.86)	1.55<br>(1.68)
Fixed Effect - KSC	-2.51<br>(4.8)	-1.88<br>(2.17)	-0.60<br>(1.18)	-0.31<br>(0.64)	-0.21<br>(0.34)	-0.21<br>(0.19)	-0.23<br>(0.16)	-0.20<br>(0.23)	-0.24<br>(0.7)	1.24<br>(1.48)
Fixed Effect - TPC	-1.50<br>(3.15)	-0.65<br>(0.98)	-0.58<br>(0.5)	-0.47<br>(0.33)	-0.26<br>(0.2)	-0.22<br>(0.13)	-0.19<br>(0.1)	-0.15<br>(0.14)	-0.62<br>(0.33)	-0.16<br>(0.8)
Elevation (m)†	0.61<br>(2.18)	0.69<br>(0.68)	0.20<br>(0.43)	0.06<br>(0.28)	0.01<br>(0.14)	-0.03<br>(0.07)	-0.05<br>(0.05)	-0.06<br>(0.08)	-0.06<br>(0.22)	0.83<br>(0.56)
Slope (degrees)	0.02<br>(0.05)	0.00<br>(0.01)	0.00<br>(0.01)	0.00<br>(0)	0.00<br>(0)	0.00<br>(0)	0.00<br>(0)	0.00<br>(0)	0.00<br>(0.01)	-0.04<br>(0.03)
Distance from road (km)	-0.13<br>(0.11)	-0.04<br>(0.07)	-0.02<br>(0.04)	-0.01<br>(0.02)	-0.01<br>(0.01)	-0.01<br>(0.01)	-0.01<br>(0)	-0.01<br>(0.01)	-0.03<br>(0.02)	-0.07<br>(0.05)
Distance from rail (km)	-0.01<br>(0.04)	-0.01<br>(0.01)	-0.01<br>(0.01)	0.00<br>(0)	0.00<br>(0)	0.00<br>(0)	0.00<br>(0)	0.00<br>(0)	-0.01<br>(0)	-0.01<br>(0.01)
Population†	-0.19<br>(0.26)	-0.03<br>(0.11)	-0.01<br>(0.07)	0.01<br>(0.05)	0.00<br>(0.03)	-0.01<br>(0.01)	-0.01<br>(0.01)	-0.01<br>(0.01)	-0.01<br>(0.04)	-0.08<br>(0.09)
Mean precipitation (mm)	0.03<br>(0.04)	0.03<br>(0.03)	0.01<br>(0.02)	0.00<br>(0.01)	0.00<br>(0.01)	0.00<br>(0)	0.00<br>(0)	0.00<br>(0)	0.00<br>(0.01)	0.00<br>(0.02)
Landholdings (ha)†	0.88<br>(0.16)	0.84<br>(0.06)	0.87<br>(0.04)	0.89<br>(0.03)	0.92<br>(0.03)	0.93<br>(0.02)	0.94<br>(0.02)	0.94<br>(0.03)	0.74<br>(0.09)	0.32<br>(0.09)
Household size	-0.15<br>(0.1)	-0.05<br>(0.04)	-0.02<br>(0.02)	-0.01<br>(0.01)	-0.01<br>(0.01)	-0.01<br>(0.01)	-0.01<br>(0.01)	0.00<br>(0.01)	0.01<br>(0.02)	0.03<br>(0.05)
Percent household female	-0.36<br>(0.61)	-0.31<br>(0.22)	-0.18<br>(0.15)	-0.10<br>(0.1)	-0.05<br>(0.06)	-0.03<br>(0.04)	-0.01<br>(0.04)	0.00<br>(0.05)	0.05<br>(0.1)	0.20<br>(0.27)

Household head education (years)	0.00<br>(0.06)	0.02<br>(0.02)	0.02<br>(0.01)	0.02<br>(0.01)	0.01<br>(0.01)	0.00<br>(0.01)	0.00<br>(0)	0.00<br>(0.01)	0.02<br>(0.01)	0.04<br>(0.03)
Household total years of education (years)	0.26<br>(0.12)	0.04<br>(0.05)	0.00<br>(0.03)	-0.01<br>(0.02)	0.01<br>(0.01)	0.01<br>(0.01)	0.01<br>(0.01)	0.01<br>(0.01)	0.02<br>(0.02)	0.03<br>(0.06)
Head age (years)†	-0.64<br>(0.57)	-0.24<br>(0.17)	-0.03<br>(0.1)	0.00<br>(0.07)	0.04<br>(0.05)	0.04<br>(0.03)	0.01<br>(0.03)	0.02<br>(0.04)	0.13<br>(0.11)	0.28<br>(0.22)
Nonfarm income (Tsh)†	0.01<br>(0.02)	0.01<br>(0.01)	0.01<br>(0.01)	0.01<br>(0)	0.00<br>(0)	0.00<br>(0)	0.00<br>(0)	0.00<br>(0)	-0.01<br>(0)	-0.01<br>(0.01)
Tropical livestock units‡	-0.22<br>(0.25)	-0.28<br>(0.25)	-0.16<br>(0.14)	-0.10<br>(0.07)	-0.07<br>(0.05)	-0.06<br>(0.03)	-0.04<br>(0.03)	-0.01<br>(0.04)	0.03<br>(0.06)	0.22<br>(0.14)
Household loan (binary)	0.12<br>(98.54)	-0.22<br>(19.27)	0.07<br>(0.29)	0.08<br>(0.2)	-0.04<br>(0.1)	-0.02<br>(0.08)	0.01<br>(12.97)	0.00<br>(65.69)	-0.01<br>(425.57)	0.50<br>(2056.37)
Total labor (hours)†	1.22<br>(0.71)	1.41<br>(0.32)	1.41<br>(0.23)	1.32<br>(0.3)	0.46<br>(0.35)	0.22<br>(0.12)	0.13<br>(0.06)	0.12<br>(0.07)	0.34<br>(0.12)	0.34<br>(0.23)
Self ag labor (hours)†	-1.59<br>(0.78)	-1.12<br>(0.33)	-1.16<br>(0.27)	-1.10<br>(0.33)	-0.34<br>(0.37)	-0.16<br>(0.13)	-0.10<br>(0.07)	-0.09<br>(0.07)	-0.27<br>(0.11)	-0.36<br>(0.31)
Household assets (count)	-0.02<br>(0.11)	-0.04<br>(0.04)	-0.03<br>(0.02)	-0.03<br>(0.02)	-0.03<br>(0.01)	-0.02<br>(0.01)	-0.02<br>(0.01)	-0.02<br>(0.01)	-0.06<br>(0.02)	-0.10<br>(0.06)

Table S5. Quantile regression model estimates of pre-acquisition (baseline) household landholdings. Bolded values are reported in Figure 2 of the main manuscript. Standard errors reported in parentheses. († = log transformed, ‡ = double-square root transformed).

term	tau (τ)									
	**0.05**	**0.15**	**0.25**	**0.35**	**0.45**	**0.55**	**0.65**	**0.75**	**0.85**	**0.95**
(Intercept)	-3.02<br>(18.34)	-1.58<br>(2.17)	-0.01<br>(0.69)	-0.89<br>(0.96)	-0.39<br>(1.14)	0.52<br>(1.58)	1.52<br>(1.71)	2.61<br>(2.55)	2.07<br>(3.31)	-1.53<br>(84.54)
**Treatment Status (β_z)**	**-0.73**<br>**(0.36)**	**-0.05**<br>**(0.12)**	**0.00**<br>**(0.03)**	**0.00**<br>**(0.03)**	**-0.01**<br>**(0.04)**	**-0.03**<br>**(0.06)**	**0.00**<br>**(0.07)**	**0.09**<br>**(0.12)**	**0.18**<br>**(0.15)**	**0.35**<br>**(0.77)**
Fixed Effect - KPL	-3.36<br>(6.66)	0.01<br>(0.97)	0.00<br>(0.23)	0.20<br>(0.37)	0.21<br>(0.49)	0.22<br>(0.59)	0.42<br>(0.65)	1.02<br>(1)	1.41<br>(1.47)	0.95<br>(43.06)
Fixed Effect - KSC	-2.78<br>(5.86)	-0.03<br>(0.81)	0.00<br>(0.18)	0.14<br>(0.3)	0.14<br>(0.38)	0.17<br>(0.45)	0.32<br>(0.5)	0.78<br>(0.77)	1.06<br>(1.21)	0.89<br>(38.39)
Fixed Effect - TPC	-0.73<br>(2.75)	0.15<br>(0.36)	0.00<br>(0.11)	0.10<br>(0.15)	0.03<br>(0.18)	-0.11<br>(0.26)	-0.18<br>(0.29)	-0.18<br>(0.39)	-0.23<br>(0.67)	0.01<br>(36.63)
Elevation (m)†	0.43<br>(2.64)	0.22<br>(0.3)	0.00<br>(0.09)	0.11<br>(0.11)	0.06<br>(0.13)	-0.05<br>(0.17)	-0.19<br>(0.19)	-0.33<br>(0.29)	-0.25<br>(0.38)	0.21<br>(11.53)
Slope (degrees)	-0.02<br>(0.05)	-0.01<br>(0.01)	0.00<br>(0)	0.00<br>(0)	0.00<br>(0)	0.00<br>(0)	-0.01<br>(0.01)	-0.01<br>(0.01)	-0.02<br>(0.01)	-0.04<br>(0.07)
Distance from road (km)	0.01<br>(0.07)	0.00<br>(0.02)	0.00<br>(0.01)	0.01<br>(0.01)	0.01<br>(0.01)	0.01<br>(0.01)	0.01<br>(0.02)	0.03<br>(0.03)	0.05<br>(0.03)	0.05<br>(0.07)
Distance from rail (km)	-0.02<br>(0.03)	0.00<br>(0.01)	0.00<br>(0)	0.00<br>(0)	0.00<br>(0)	0.00<br>(0)	0.00<br>(0)	0.01<br>(0)	0.01<br>(0.01)	0.00<br>(0.07)
Population†	-0.16<br>(0.19)	-0.03<br>(0.05)	0.00<br>(0.02)	0.00<br>(0.02)	0.00<br>(0.03)	-0.01<br>(0.03)	-0.03<br>(0.04)	-0.02<br>(0.05)	0.02<br>(0.07)	0.01<br>(0.2)
Mean precipitation (mm)	0.03<br>(0.04)	0.00<br>(0.01)	0.00<br>(0)	0.00<br>(0)	0.00<br>(0.01)	0.00<br>(0.01)	-0.01<br>(0.01)	-0.01<br>(0.01)	-0.02<br>(0.01)	-0.01<br>(0.1)
Farm size (ha)†	0.72<br>(0.09)	0.97<br>(0.04)	1.00<br>(0.03)	0.97<br>(0.07)	0.82<br>(0.13)	0.64<br>(0.13)	0.59<br>(0.1)	0.55<br>(0.09)	0.47<br>(0.06)	0.36<br>(0.09)
Household size	0.00<br>(0.09)	0.00<br>(0.02)	0.00<br>(0.01)	0.01<br>(0.01)	0.03<br>(0.02)	0.03<br>(0.02)	0.04<br>(0.02)	0.06<br>(0.03)	0.07<br>(0.04)	0.03<br>(0.08)
	-0.25	-0.08	0.00	-0.03	0.01	0.02	0.12	0.22	0.34	0.36

Percent household female	(0.44)	(0.12)	(0.04)	(0.05)	(0.07)	(0.08)	(0.12)	(0.17)	(0.21)	(0.33)
Household head education (years)	-0.02<br>(0.05)	0.00<br>(0.01)	0.00<br>(0)	0.00<br>(0)	0.00<br>(0.01)	0.01<br>(0.01)	0.01<br>(0.01)	0.01<br>(0.01)	0.03<br>(0.02)	0.05<br>(0.03)
Household total years of education (years)	0.00<br>(0.11)	0.00<br>(0.03)	0.00<br>(0.01)	-0.01<br>(0.01)	-0.01<br>(0.02)	-0.03<br>(0.02)	-0.03<br>(0.03)	-0.01<br>(0.03)	-0.02<br>(0.04)	-0.01<br>(0.08)
Head age (years)†	0.45<br>(0.44)	0.02<br>(0.12)	0.00<br>(0.03)	0.04<br>(0.04)	0.04<br>(0.06)	0.13<br>(0.08)	0.22<br>(0.1)	0.32<br>(0.13)	0.48<br>(0.17)	0.64<br>(0.34)
Nonfarm income (Tsh)†	-0.02<br>(0.02)	0.00<br>(0.01)	0.00<br>(0)	0.00<br>(0)	0.00<br>(0)	-0.01<br>(0)	-0.01<br>(0)	-0.01<br>(0)	-0.01<br>(0.01)	-0.02<br>(0.01)
Tropical livestock units‡	0.06<br>(0.19)	0.01<br>(0.06)	0.00<br>(0.03)	0.05<br>(0.04)	0.10<br>(0.06)	0.16<br>(0.08)	0.20<br>(0.09)	0.21<br>(0.1)	0.24<br>(0.11)	0.24<br>(0.19)
Household loan (binary)	0.53<br>(97.47)	0.07<br>(22.42)	0.02<br>(13.17)	0.02<br>(0.07)	0.09<br>(0.1)	0.15<br>(0.13)	0.21<br>(0.17)	0.23<br>(0.25)	0.24<br>(37.1)	0.24<br>(1641.98)
Total labor (hours)†	0.17<br>(0.27)	0.02<br>(0.06)	0.00<br>(0.03)	-0.05<br>(0.07)	-0.12<br>(0.14)	-0.39<br>(0.16)	-0.29<br>(0.22)	-0.28<br>(0.22)	-0.23<br>(0.14)	0.13<br>(0.3)
Self ag labor (hours)†	-0.24<br>(0.37)	-0.04<br>(0.08)	0.00<br>(0.04)	0.04<br>(0.08)	0.10<br>(0.15)	0.34<br>(0.18)	0.22<br>(0.22)	0.19<br>(0.24)	0.07<br>(0.21)	-0.27<br>(0.46)
Household assets (count)	-0.16<br>(0.08)	-0.02<br>(0.03)	0.00<br>(0.01)	-0.01<br>(0.01)	-0.02<br>(0.02)	-0.05<br>(0.03)	-0.05<br>(0.03)	-0.08<br>(0.03)	-0.10<br>(0.03)	-0.10<br>(0.06)

Table S6. Regression tables for land value estimates using i) landholdings determined as all the land parcels identified by respondents and ii) farm size using only cultivated land parcels. Land values are normalized by land area and estimated as 2013 USD per hectare (USD/ha)

	Land Value (USD/ha)	
	Landholdings	Farm Size
	(1)	(2)
Year dummy (2013)	0.158***	0.026
	(0.059)	(0.065)
LSLA distance (km)	-0.013***	-0.014***
	(0.005)	(0.003)
Farm size dummy (>50th percentile)	-0.190	-0.261
	(0.529)	(0.352)
LSLA distance * Farm size dummy	0.002	0.004***
	(0.004)	(0.001)
Year dummy * LSLA distance	0.002	0.003**
	(0.002)	(0.001)
Farm size dummy * Wave dummy	-0.610	-0.333
	(0.405)	(0.282)
Distance from home (km)	-0.053	-0.036
	(0.059)	(0.045)
Distance from road (km)	-0.013	-0.021
	(0.015)	(0.015)
Distance from market (km)	-0.009	-0.005
	(0.007)	(0.005)
Good soil	0.437***	0.327***
	(0.147)	(0.081)
Bad soil	-0.676	-0.621
	(0.796)	(0.795)
Flat	-0.335***	-0.268
	(0.026)	(0.184)
Steep	-0.142	-0.209
	(0.476)	(0.391)
Formal title	-0.246	-0.170
	(0.265)	(0.116)
Informal title	0.248***	0.104***
	(0.070)	(0.004)
Manyara Region (FE)	-1.200***	-1.207***
	(0.149)	(0.183)
Morogoro Region (FE)	-1.322***	-1.391***
	(0.363)	(0.286)
Constant	8.820***	8.837***
	(0.357)	(0.309)

Notes: ***Significant at the 1 percent level.
**Significant at the 5 percent level.
*Significant at the 10 percent level.

Table S7. Marginal effect of pre-acquisition household income on land asset change. († = log transformed).

Pre-Acquisition Income (Tsh)†	*Farm Size Change*		*Landholdings Change*	
	**Marginal Treatment Effect**	**Standard Error**	**Marginal Treatment Effect**	**Standard Error**
9.62	-3.56	2.18	-2.59	1.58
9.79	-3.45	2.09	-2.53	1.51
9.97	-3.33	2.01	-2.47	1.45
10.15	-3.17	1.92	-2.34	1.38
10.32	-3.01	1.80	-2.22	1.29
10.50	-2.81	1.67	-2.05	1.19
10.68	-2.57	1.52	-1.82	1.06
10.86	-2.36	1.39	-1.64	0.96
11.03	-2.22	1.29	-1.55	0.89
11.21	-2.09	1.21	-1.48	0.83
11.39	-1.94	1.11	-1.36	0.77
11.57	-1.77	1.00	-1.24	0.69
11.74	-1.59	0.90	-1.11	0.63
11.92	-1.42	0.80	-0.99	0.57
12.10	-1.23	0.69	-0.87	0.51
12.27	-1.02	0.59	-0.75	0.45
12.45	-0.83	0.50	-0.65	0.42
12.63	-0.67	0.44	-0.60	0.39
12.81	-0.55	0.40	-0.57	0.37
12.98	-0.47	0.39	-0.55	0.36
13.16	-0.43	0.38	-0.55	0.35
13.34	-0.40	0.39	-0.55	0.36
13.52	-0.37	0.41	-0.54	0.37
13.69	-0.34	0.42	-0.51	0.39
13.87	-0.31	0.44	-0.48	0.40
14.05	-0.29	0.45	-0.45	0.42
14.22	-0.26	0.46	-0.41	0.42
14.40	-0.20	0.47	-0.37	0.42
14.58	-0.09	0.51	-0.32	0.41
14.76	0.06	0.57	-0.23	0.42
14.93	0.24	0.66	-0.12	0.43
15.11	0.42	0.76	0.01	0.46
15.29	0.64	0.88	0.17	0.53
15.47	0.86	1.02	0.34	0.62
15.64	1.06	1.16	0.50	0.72
15.82	1.26	1.29	0.65	0.83
16.00	1.48	1.44	0.82	0.96

16.17	1.71	1.58	1.00	1.09
16.35	1.92	1.72	1.19	1.22
16.53	2.13	1.86	1.35	1.34
16.71	2.33	1.99	1.52	1.46
16.88	2.53	2.11	1.69	1.58
17.06	2.73	2.23	1.87	1.69
17.24	2.92	2.35	2.05	1.79
17.42	3.10	2.46	2.22	1.88
17.59	3.28	2.58	2.38	1.98
17.77	3.45	2.70	2.52	2.08
17.95	3.63	2.82	2.64	2.18
18.12	3.82	2.95	2.76	2.29
18.30	3.99	3.07	2.89	2.39

Table S8. Marginal treatment effect of pre-acquisition household landholdings on land asset change. († = log transformed).

Pre-Acquisition Landholdings†	Farm Size Change		Landholdings Change	
	Marginal Treatment Effect	Standard Error	Marginal Treatment Effect	Standard Error
-4.61	2.02	1.69	0.72	0.86
-4.42	1.82	1.55	0.56	0.82
-4.23	1.58	1.43	0.47	0.94
-4.04	1.38	1.42	0.44	1.11
-3.85	1.24	1.43	0.42	1.21
-3.66	1.14	1.42	0.40	1.23
-3.48	1.07	1.38	0.39	1.20
-3.29	1.02	1.31	0.37	1.13
-3.10	0.98	1.23	0.35	1.04
-2.91	0.93	1.12	0.32	0.93
-2.72	0.84	1.00	0.25	0.77
-2.53	0.71	0.86	0.14	0.59
-2.35	0.56	0.75	0.05	0.44
-2.16	0.44	0.68	0.00	0.40
-1.97	0.39	0.66	0.00	0.43
-1.78	0.32	0.62	-0.02	0.42
-1.59	0.21	0.55	-0.07	0.36
-1.41	0.14	0.52	-0.09	0.35
-1.22	0.08	0.49	-0.11	0.34
-1.03	-0.04	0.43	-0.17	0.30
-0.84	-0.15	0.39	-0.22	0.27
-0.65	-0.20	0.38	-0.25	0.27
-0.46	-0.22	0.38	-0.27	0.27
-0.28	-0.20	0.38	-0.27	0.28
-0.09	-0.15	0.40	-0.27	0.30
0.10	-0.13	0.44	-0.27	0.33
0.29	-0.13	0.48	-0.30	0.37
0.48	-0.15	0.51	-0.33	0.41
0.67	-0.15	0.56	-0.31	0.48
0.85	-0.07	0.63	-0.21	0.57
1.04	0.06	0.73	-0.07	0.68
1.23	0.19	0.83	0.05	0.78
1.42	0.32	0.93	0.16	0.88
1.61	0.40	1.02	0.25	0.97
1.79	0.42	1.09	0.29	1.04
1.98	0.39	1.15	0.31	1.10
2.17	0.38	1.21	0.33	1.16

2.36	0.38	1.28	0.36	1.23
2.55	0.38	1.35	0.38	1.30
2.74	0.36	1.41	0.39	1.36
2.92	0.32	1.46	0.39	1.42
3.11	0.28	1.53	0.39	1.49
3.30	0.23	1.60	0.39	1.56
3.49	0.11	1.63	0.35	1.60
3.68	-0.07	1.63	0.28	1.62
3.86	-0.26	1.64	0.20	1.63
4.05	-0.36	1.68	0.17	1.68
4.24	-0.28	1.78	0.22	1.78
4.43	-0.13	1.92	0.30	1.90
4.62	-0.05	2.05	0.36	2.01

Table S9. Marginal effect of pre-acquisition household labor on land asset change. († = log transformed).

Pre-Acquisition Labor (Tsh)†	*Farm Size Change*		*Landholdings Change*	
	**Marginal Treatment Effect**	**Standard Error**	**Marginal Treatment Effect**	**Standard Error**
0.00	-2.02	1.65	-0.45	1.42
0.05	-2.03	1.63	-0.45	1.40
0.09	-2.10	1.65	-0.44	1.41
0.14	-2.12	1.65	-0.44	1.42
0.18	-2.08	1.62	-0.43	1.39
0.23	-2.05	1.59	-0.42	1.38
	-2.03	1.57	-0.41	1.36
0.32	-1.99	1.54	-0.40	1.35
0.37	-1.94	1.50	-0.39	1.32
0.41	-1.86	1.45	-0.37	1.29
0.46	-1.78	1.39	-0.35	1.25
0.50	-1.70	1.33	-0.33	1.21
0.55	-1.62	1.28	-0.32	1.18
0.60	-1.55	1.23	-0.31	1.14
0.64	-1.48	1.19	-0.29	1.11
0.69	-1.42	1.15	-0.27	1.09
0.73	-1.35	1.11	-0.25	1.06
0.78	-1.28	1.07	-0.22	1.03
0.83	-1.20	1.02	-0.21	1.00
0.87	-1.14	0.98	-0.20	0.96
0.92	-1.08	0.93	-0.20	0.93
0.96	-1.02	0.89	-0.20	0.90
1.01	-0.96	0.85	-0.19	0.87
1.06	-0.89	0.82	-0.16	0.85
1.10	-0.82	0.79	-0.15	0.82
1.15	-0.75	0.76	-0.13	0.80
1.19	-0.70	0.73	-0.13	0.78
1.24	-0.68	0.71	-0.15	0.75
1.28	-0.66	0.69	-0.17	0.73
1.33	-0.64	0.67	-0.19	0.71
1.38	-0.65	0.66	-0.26	0.70
1.42	-0.60	0.65	-0.30	0.69
1.47	-0.48	0.65	-0.27	0.69
1.51	-0.33	0.67	-0.19	0.69
1.56	-0.18	0.71	-0.09	0.70
1.61	-0.02	0.76	0.02	0.72

1.65	0.16	0.82	0.14	0.75
1.70	0.36	0.91	0.27	0.79
1.74	0.56	0.98	0.39	0.82
1.79	0.76	1.05	0.51	0.84
1.84	0.97	1.11	0.62	0.85
1.88	1.14	1.16	0.70	0.85
1.93	1.30	1.22	0.77	0.86
1.97	1.46	1.29	0.83	0.88
2.02	1.60	1.35	0.87	0.89
2.06	1.64	1.38	0.82	0.88
2.11	1.54	1.36	0.70	0.85
2.16	1.41	1.32	0.59	0.83
2.20	1.37	1.32	0.55	0.83
2.25	1.43	1.35	0.55	0.84

Table S10. Mediation analysis of well-being outcomes conditional on LSLA treatment and farmland decreases using weighted linear regression. The mediation analysis includes two stages with stage one estimating the effect of LSLA treatment on decreases in household farmland. The second stage estimates the influence of farmland decreases in treatment and control household for four well-being measures including total income, wealth, the multi-dimensional poverty index (MPI) and food insecurity. Bolded values are reported in Figure 4 of the main manuscript. Standard errors reported in parentheses. Significance levels denoted by *** p < 0.001; ** p < 0.01; * p < 0.05. Data transformations denoted by † = log transformed; ‡ = double-square root transformed.

	Stage One	Stage Two			
	**Farm decrease†**	**Total income (Tsh)**	**Wealth index**	**MPI**	**Food insecurity**
**Treatment Status (β_z)**	**0.13 ***	0.07	0.09	0.01	-0.02
	**(0.07)**	(0.16)	(0.11)	(0.01)	(0.03)
**Farmland decrease - binary (γ)**		**0.03**	**-0.06**	**0.01**	**0.07**
		**(0.17)**	**(0.21)**	**(0.01)**	**(0.06)**
Treatment * Farmland decrease (δ)		-0.20	-0.31	0.01	0.05
		(0.23)	(0.23)	(0.01)	(0.08)
**Linear combination ($\gamma + \delta$)**		**-0.17**	**-0.37 ****	**0.03 ***	**0.12 ***
		**(0.24)**	**(0.13)**	**(0.01)**	**(0.05)**
Elevation (m) †	0.04	-0.00	-0.40	0.06 **	-0.03
	(0.08)	(0.51)	(0.33)	(0.02)	(0.11)
Slope (degrees)	-0.01	0.00	-0.02 **	0.00 ***	0.00
	(0.00)	(0.02)	(0.01)	(0.00)	(0.00)
Distance from road (km)	-0.01	0.01	0.01	-0.00	0.00
	(0.01)	(0.05)	(0.03)	(0.00)	(0.01)
Distance from rail (km)	0.00	0.00	-0.00	-0.00 *	-0.00
	(0.00)	(0.01)	(0.00)	(0.00)	(0.00)
Population †	0.02	0.08	0.23 **	-0.02 **	-0.00
	(0.02)	(0.08)	(0.07)	(0.01)	(0.02)
Mean precipitation	-0.01 **	-0.01	-0.01	0.00	0.00
	(0.00)	(0.01)	(0.01)	(0.00)	(0.00)
Baseline farm size (ha)	0.07 ***	0.01	0.06	0.00	-0.02
	(0.01)	(0.06)	(0.03)	(0.00)	(0.01)
Baseline landholdings (ha)	-0.00	0.02	0.06	-0.00	-0.01
	(0.01)	(0.07)	(0.03)	(0.00)	(0.02)
Household size	-0.03 ***	0.23 ***	0.09 **	-0.00	-0.00
	(0.01)	(0.06)	(0.03)	(0.00)	(0.01)
Percent household female	0.10	0.80	0.53 **	-0.05 **	-0.03
	(0.06)	(0.41)	(0.19)	(0.02)	(0.06)

	Stage One	Stage Two			
	Farm decrease†	Total income (Tsh)	Wealth index	MPI	Food insecurity
Household head education (years)	-0.01 *	0.05	0.10 ***	-0.01 ***	-0.03 ***
	(0.00)	(0.04)	(0.02)	(0.00)	(0.01)
Household total years of education (years)	0.01	-0.11	-0.03	-0.01 **	0.01
	(0.01)	(0.05)	(0.04)	(0.00)	(0.01)
Head age (years)†	0.17 ***	-0.63 *	-0.08	0.03 **	0.06
	(0.04)	(0.28)	(0.16)	(0.01)	(0.04)
Nonfarm income (Tsh)†	0.00		-0.01	0.00 **	0.01 *
	(0.00)		(0.01)	(0.00)	(0.00)
Tropical livestock units‡	0.01	0.63 ***	-0.01	-0.01	-0.05
	(0.02)	(0.11)	(0.08)	(0.01)	(0.03)
Household loan (binary)	-0.03	0.23	0.57 *	-0.03	-0.03
	(0.09)	(0.24)	(0.24)	(0.02)	(0.06)
Total labor (hours)†	-0.05	0.33	0.30	0.01	-0.02
	(0.05)	(0.33)	(0.17)	(0.02)	(0.06)
Self ag labor (hours)†	-0.01	-0.45 *	-0.43 ***	0.00	-0.00
	(0.03)	(0.20)	(0.09)	(0.01)	(0.03)
Household assets (count)	0.01	-0.40 ***	-0.11 ***	0.02 ***	0.06 ***
	(0.01)	(0.07)	(0.03)	(0.00)	(0.01)
Fixed Effect - KPL	0.84	1.49	-0.12	-0.07	-0.17
	(0.46)	(1.49)	(0.58)	(0.09)	(0.24)
Fixed Effect - KSC	0.69 *	0.38	0.34	-0.09	-0.08
	(0.35)	(1.11)	(0.46)	(0.08)	(0.20)
Fixed Effect - TPC	0.10	-0.46	-0.11	-0.06	-0.04
	(0.18)	(0.66)	(0.48)	(0.04)	(0.15)
Intercept	-0.27	20.39 ***	6.41 *	-0.50 **	-0.26
	(0.73)	(3.34)	(2.84)	(0.18)	(0.90)

Table S11. List of large-scale land acquisitions compiled from government reports and literature. We include only sites where geo-location data was available at the time of survey design.

S/N	Previous owner	Year	Administrative Location		Size (ha)	Purpose of investment	Investing institution	Selected for this study
			Region	*District*				
1	National Agricultural & Food Corporation (NAFCO)	2000	Morogoro	Kilombero	12,298	Sugar cane	Kilombero Sugar Company (Illovo Sugar Co.)	Yes
2	National Agricultural & Food Corporation (NAFCO)	2000	Kilimanjaro	Moshi Rural	10,349	Sugar cane	Tanganyika Plantation Company (Alteo Ltd.)	Yes
3	National Agricultural & Food Corporation (NAFCO)	2005	Manyara	Hanang	17,790	Wheat production	Ngano Ltd (Hanang Wheat Complex)	Yes
4	National Agricultural & Food Corporation (NAFCO)	2005	Manyara	Hanang	13,725	Wheat production	Hydom Lutheran Hospital and Development Ltd	No
5	National Agricultural & Food Corporation (NAFCO)	2005	Manyara	Hanang	13,583	Wheat production	Hanang district council	No
6	National Agricultural & Food Corporation (NAFCO)	2006	Mbeya	Mbarari	7,370	Rice production	Export Ltd Trading Co.	No
7	National Agricultural & Food Corporation (NAFCO)	2006	Mbeya	Mbarari	6,030	Rice production	Highland Estate Co. Ltd	No
8	National Agricultural & Food Corporation (NAFCO)	2006	Pwani	Bagamoyo	3,209	Rice production	Bagamoyo district council	No
9	National Agricultural & Food Corporation (NAFCO)	2006	Morogoro	Mvomero	2,000	Rice production	Mvomero district council	No
10	Tanzania Sisal Authority (TSA)	2007	Tanga	Tanga Urban	5,537	Sisal production	Coal Tail Enterprise Ltd	No

11	National Agricultural & Food Corporation (NAFCO)	2009	Mbeya	Mbozi	4,842	Seeds production	Agriculture Seed Agency	No
12	Tanzania Sisal Authority (TSA)	2014	Tanga	Tanga Urban	2,000	Sisal production	Marungu Estate Ltd	No
13	Ndundu/Nyamwage villages	2005	Coast Region	Rufiji	3,060	Agriculture	Safe Agricultural Products Ltd	No
14	Basanza village	2008	Kigoma	Uvinza	4,257	Palm oil	Felia Coy Ltd	No
15	Unknown	2008	Morogoro	Kilombero	5,158	Rice production	Kilombero Plantation Ltd (Agrica Ltd.)	Yes
16	Lipokela village	2013	Ruvuma	Songea	1,999	Coffee	Aviv Limited	No
17	Kilimani Village	2013	Coast Region	Rufiji	1,301	Agriculture	Euro Vista Ltd	No
18	Kipugira village	2015	Coast Region	Rufiji	5,506	Agriculture	Lukuliro Farm Holdings Ltd	No
19	Unknown	2009	Tanga	Mkinga	5,386	Agriculture	Arkadia Company Ltd.	No
20	Unknown	2013	Mara	Serengeti	6,015	Wheat production	Mountainside Farms Company Ltd.	No
21	Unknown	2013	Kigoma	Kasulu	48,017	Sugar cane	Tanzania Investment Center	No
22	Unknown	2013	Morogoro	Kilombero	4,205	Agriculture	Tanzania Investment Center	No
23	Unknown	2014	Lindi	Lindi	536	Cassava	Cassav Starch of Tanzania Corporation Ltd	No
24	Unknown	2014	Pwani	Kisarawe	4,000	Agriculture	National Development Cooperation (NDC)	No
25	Unknown	2014	Tanga	Mkinga	1,321	Agriculture	Mavovo Farm Ltd	No

Table S12. Summary of selected villages for our household survey instrument categorized by large-scale land acquisition site and treatment status. Sampled households per village ranged from 16 to 52.

LSLA Site Name	Village Name	Treatment Status	Sample size (n)
Hanang Wheat Complex	Gidihim	Control	33
Hanang Wheat Complex	Maretadu Chini	Control	29
Hanang Wheat Complex	Simhha	Control	30
Hanang Wheat Complex	Dajameda	Treatment	31
Hanang Wheat Complex	Galangal	Treatment	29
Hanang Wheat Complex	Muungano	Treatment	32
Kilombero Plantation Ltd.	Mbingu	Control	51
Kilombero Plantation Ltd.	Vigaeni	Control	52
Kilombero Plantation Ltd.	Isago	Treatment	35
Kilombero Plantation Ltd.	Itongowa	Treatment	35
Kilombero Plantation Ltd.	Mkangawalo	Treatment	30
Kilombero Sugar Company	Ihombwe	Control	36
Kilombero Sugar Company	Lumango	Control	23
Kilombero Sugar Company	Mikumi	Control	31
Kilombero Sugar Company	Msimba	Control	34
Kilombero Sugar Company	Chicago	Treatment	27
Kilombero Sugar Company	Kidatu Kati	Treatment	24
Kilombero Sugar Company	Kidogobasi	Treatment	16
Kilombero Sugar Company	Mkamba	Treatment	23
Kilombero Sugar Company	Msolwa Ujamaa	Treatment	17
Kilombero Sugar Company	Msowero	Treatment	21
Kilombero Sugar Company	Nyange	Treatment	24
Kilombero Sugar Company	Ruhembe	Treatment	22
Tanganyika Plantation Company	Handeni	Control	31
Tanganyika Plantation Company	Kisangiro	Control	25
Tanganyika Plantation Company	Kyomu	Control	30
Tanganyika Plantation Company	Langata Bora	Control	25
Tanganyika Plantation Company	Mandaka	Control	23
Tanganyika Plantation Company	Mforo	Control	24
Tanganyika Plantation Company	Chekereni Weruweru	Treatment	27
Tanganyika Plantation Company	Matindigani	Treatment	27
Tanganyika Plantation Company	Mikocheni	Treatment	26
Tanganyika Plantation Company	Mserekia	Treatment	25
Tanganyika Plantation Company	Mtakuja	Treatment	31
Tanganyika Plantation Company	Oria	Treatment	24

Table S13. Geographic covariates used in our region growing algorithm to generate a control zone for which control villages were subsequently selected during fieldwork in 2017 and 2018.

Description	Resolution (m)	Year	Units	Citation
**Time Independent**				
Elevation	30	n/a	meters	SRTM 30m[25]
Slope	30	n/a	percent slope	SRTM 30m[25]
Soil productivity	250	n/a	index (1-18)	Schaetzl et al (2012)[26]
Distance from road	30	n/a	meters	OpenStreetMap[27]
Distance from railway	30	n/a	meters	OpenStreetMap[27]
Distance from Protected Area	30	n/a	meters	WDPA[28]
Average annual temperature	1000	1970-2000	Celsius	WorldClim2[29]
Average annual precipitation	1000	1970-2000	millimeters	WorldClim2[29]
**Time Varying**				
Forest Cover	30	2000-2015	indicator (0/1)	Global Forest Change[30]
Population density	100	2000-2017	ppl/ha^2	WorldPop[31]

Table S14. Household sampling intensity was estimated from baseline population estimates and household size. For each site we targeted at least 100 household surveys or a 2.5% sampling rate.

	LSLA Site			
**LSLA Site**	**Hanang Wheat Complex**	**Kilombero Plantation Ltd.**	**Kilombero Sugary Company**	**Tanganyika Plantation Company**
Estimated Population (Baseline)[31]	21,873	6,323	46,145	49,406
Household Size (2012)	5.9	4.7	3.9	4.1
Estimated Number of Households	3,707	1,345	11,832	12,050
Sampling Intensity	3.0%	8.0%	2.5%	2.5%
Target Number of Surveys	111	108	296	301
Realized Number of Surveys	184	203	298	318

Table S15. Summary of village population at the time of survey collection using household rosters. Eligible households represent those that resided in a village prior to the start of LSLAs and thus can provide retrospective information. Ineligible households represent new residents and thus provide estimates of in-migration.

Site	Status	Avg. Elgibile HH	Avg. Ineligible HH	Perc. Ineligible	Avg. # of Households	Avg. Population
Hanang Wheat Complex	Treatment	125	329	72%	454	2356
	Control	236	105	31%	341	1847
Kilombero Sugar Company	Treatment	526	663	56%	1189	5167
	Control	350	455	57%	805	2991
Kilombero Plantation	Treatment	366	204	36%	569	2356
	Control	180	541	75%	720.5	3061.5
Tanganyika Plantation Co.	Treatment	190	900	83%	1090	6540
	Control	161	220	58%	381	1419

Table S16. Measures of covariate balance before and after adjustment using entropy balance. Calculations for standardized mean differences use pooled standard deviations for estimating the Quantile Treatment Effect (QTE). Standard deviations are reported in parentheses and represent variation across household survey imputations. († = log transformed).

Source	Variable	Mean (S.D.) - Control	Mean (S.D.) - Treatment	Standardized Mean Difference	
				Unadjusted	*Adjusted*
SRTM 30m	Elevation (m)†	6.49 (0.62)	6.29 (0.68)	-0.31	<0.0001
	Slope (%)	5.14 (3.82)	4.36 (4.4)	-0.19	<0.0001
Open Street Map	Distance from road (km)	2.47 (2.51)	1.90 (2.33)	-0.24	<0.0001
	Distance from rail (km)	26.24 (32.77)	16.22 (20.18)	-0.37	<0.0001
WorldPop	Population density (persons/ha)†	-0.28 (1.03)	0.12 (1.37)	0.34	<0.0001
WorldClim	Mean precipitation (mm)	81.77 (35.29)	88.90 (36.59)	0.20	<0.0001
Household Survey	Baseline landholdings (ha)†	-0.19 (1.48)	-0.41 (1.63)	-0.14	<0.0001
	Baseline farm size (ha)†	-0.73 (1.95)	-0.67 (1.88)	0.03	<0.0001
	Household size (persons)	3.89 (1.99)	4.05 (2.04)	0.08	<0.0001
	Household percent female (%)	0.44 (0.24)	0.44 (0.22)	-0.03	<0.0001
	Household education (years)	6.11 (2.87)	5.85 (3.16)	-0.08	<0.0001
	Household head education (years)	1.40 (1.65)	1.76 (1.8)	0.21	<0.0001
	Household head age (years)†	3.68 (0.31)	3.65 (0.31)	-0.10	<0.0001
	Nonfarm Income (Tsh)†	4.26 (6.44)	5.64 (6.94)	0.21	<0.0001
	Livestock (TLU) ^ 0.25	0.54 (0.71)	0.39 (0.68)	-0.22	<0.0001
	Household loan (1=Yes)	0.03 --	0.03 --	0.03	<0.0001
	Total Household Labor (hours/week)†	1.64 (0.3)	1.62 (0.39)	-0.05	<0.0001
	Household Farm Labor (hours/week)†	1.26 (0.63)	1.28 (0.61)	0.03	<0.0001
	Household assets (count)	10.85 (1.43)	10.70 (1.68)	-0.10	<0.0001

Table S17. Summary of each well-being outcome and the household survey inputs used to construct them. Household survey questions spanned elements of income, education, health, living standards, asset ownership, housing materials, and finance to represent varying elements of well-being.

**Dimension**	**Household Survey Question**	**Well-being Outcomes**			
		**Total Income**	**MPI**	**Wealth Index**	**Food Insecurity**
**Income**	How much cash and non-cash income did the household receive in the last year from each of the following sources: sale of crops, livestock, livestock products, on-farm wage labor, off-farm wage labor, off-farm employment, off-farm business income, remittances, fishing, timber sales, rent for land, government support or other (reported in Tanzania Shilling [TZS])	Sum of income sources (log transformed)			
**Education**	How many household members are educated above 5 years of formal education?		No household member has completed five years of schooling.		
	Do all children in this household, above 4 and below 15 years of age, attend school almost every day (4-5 days a week) while school is in session?		Any school-aged child is not attending school.		
**Health**	How many household member(s) died in the last year?		Any child has died in the last year.**		
	How many days in the last year did you have problems satisfying the food needs of your household?		Any adult or child for whom there is nutritional information is malnourished (> 30 days [binary])		> 0 days (binary)
**Living Standards**	Does this household have electricity?		The household has no electricity.	Yes - binary	
	What kind of toilet facility do members of your household usually use? Do you share this toilet facility with other household(s)?		The household's sanitation facility is not improved , or it is improved but	Access to improve sanitation (binary)	

			shared with other households.		
	What are your household's top two primary sources of fuel for cooking?		The household cooks with dung, wood or charcoal.		
	What is the main source of drinking water for members of your household? If primary water source is not piped to the house, how far away is the water source?		The household does not have access to safe drinking water or safe drinking water is more than a 30-minute walk from home roundtrip.	Access to safe drinking water	
**Assets Ownership**	Please indicate if your household owns/owned any of the following assets: radio, phone, cellphone, television, fridge, stove, car, motobike, bicycle, tractor, house, plow		The household does not own more than one radio, TV, telephone, bike, motorbike or refrigerator and does not own a car or truck.	All assets as binary indicators	
**Household building materials**	What is the floor made of?		The household has a dirt, sand or dung floor.	Non-dirt floor - binary	
	What is the roof made of?			Metal or tile - binary	
	What are the walls made of?			Brick, concrete or metal - binary	
**Savings account**	Does a member of your household have an account at a bank, micro finance institution, mobile money, village savings organization or another financial institution?			Yes - binary	

Table S18. Comparison of landholdings and farm size between the National Panel Survey implemented by the Tanzania National Bureau of Statistics and our household survey across quantiles. We draw from National Panel Survey wave 1 (2009) and wave 4 (2015) to compare to our household survey for baseline and 2018 years, respectively.

	Quantiles									
**Landholdings**	**0-5%**	**15%**	**25%**	**35%**	**45%**	**55%**	**65%**	**75%**	**85%**	**95%**
LSMS Wave 1 (2008/2009)	0.14	0.38	0.59	0.87	1.14	1.47	1.92	2.44	3.61	6.89
Our HH Survey (Baseline)	0.00	0.20	0.41	0.61	0.81	1.20	1.42	1.80	2.64	5.41
Percent Difference	NA	61.2%	37.0%	35.6%	33.9%	20.1%	30.1%	30.3%	31.1%	24.0%
LSMS Wave 4 (2014/2015)	0.12	0.40	0.53	0.81	0.97	1.21	1.62	2.23	3.36	6.79
Our HH Survey (2018)	0.10	0.20	0.41	0.81	0.98	1.22	1.62	2.03	3.24	6.21
Percent Difference	19.3%	66.6%	26.0%	0.4%	0.5%	0.1%	0.1%	9.4%	3.6%	8.8%
**Farm Size**	**5%**	**15%**	**25%**	**35%**	**45%**	**55%**	**65%**	**75%**	**85%**	**95%**
LSMS Wave 1 (2008/2009)	0.14	0.33	0.53	0.76	1.04	1.32	1.70	2.34	3.43	6.16
Our HH Survey (Baseline)	0.00	0.00	0.40	0.60	0.81	0.81	1.22	1.62	2.30	4.31
Percent Difference	NA	NA	28.6%	23.9%	25.3%	47.8%	33.4%	36.4%	39.5%	35.5%
LSMS Wave 4 (2014/2015)	0.12	0.36	0.49	0.73	0.89	1.21	1.62	2.05	3.24	6.24
Our HH Survey (2018)	0.00	0.20	0.41	0.61	0.81	1.01	1.22	1.62	2.44	6.02
Percent Difference	NA	58.2%	18.1%	18.1%	9.4%	18.1%	28.5%	23.6%	28.2%	3.5%

References

1. Sullivan, J. A., Brown, D. G., Moyo, F., Jain, M. & Agrawal, A. Impacts of large-scale land acquisitions on smallholder agriculture and livelihoods in Tanzania. *Environ. Res. Lett.* **17**, (2022).
2. Deininger, K. & Xia, F. Quantifying Spillover Effects from Large Land-based Investment: The Case of Mozambique. *World Dev.* **87**, 227–241 (2016).
3. Jung, S., Liao, C., Agrawal, A. & Brown, D. G. Evidence on Wealth-Improving Effects of Forest Concessions in Liberia. *J. Assoc. Environ. Resour. Econ.* **6**, 961–998 (2019).
4. Bluwstein, J. *et al.* Between dependence and deprivation: The interlocking nature of land alienation in Tanzania. *J. Agrar. Chang.* **18**, 806–830 (2018).
5. Grossman, G. & Lewis, J. I. Administrative unit proliferation. *Am. Polit. Sci. Rev.* **108**, 196–217 (2014).
6. Bluwstein, J. *et al.* A quasi- experimental study of impacts of Tanzania's wildlife management areas on rural livelihoods and wealth. *Sci. Data* 1–13 (2018).
7. Lloyd, C. T. High resolution global gridded data for use in population studies. *Int. Arch. Photogramm. Remote Sens. Spat. Inf. Sci. - ISPRS Arch.* **42**, 117–120 (2017).
8. National Bureau of Statistics. *2012 Population and housing census; Population Distribution by Adminstrative Areas*. *National Bureau of Statistics* (2013).
9. Godlonton, S., Hernandez, M. A. & Murphy, M. Anchoring Bias in Recall Data: Evidence from Central America. *Am. J. Agric. Econ.* **100**, 479–501 (2018).
10. Keane, A. *et al.* Impacts of Tanzania's Wildlife Management Areas on household wealth. *Nat. Sustain.* (2019) doi:10.1038/s41893-019-0458-0.
11. Sills, E. O. *et al.* Building the evidence base for REDD+: Study design and methods for evaluating the impacts of conservation interventions on local well-being. *Glob. Environ. Chang.* **43**, 148–160 (2017).
12. Jagger, P., Luckert, M. K., Banana, A. & Bahati, J. Asking Questions to Understand Rural Livelihoods: Comparing Disaggregated vs. Aggregated Approaches to Household Livelihood Questionnaires. *World Dev.* (2012) doi:10.1016/j.worlddev.2012.04.030.
13. Ginn, J. & Silge, J. qualtRics: Download 'Qualtrics' Survey Data. (2020).
14. van Buuren, S. & Groothuis-Oudshoorn, K. mice: Multivariate Imputation by Chained Equations in R. *J. Stat. Softw.* **45**, 1–67 (2011).
15. Bebbington, A. Capitals and Capabilities, A Framework for Analyzing and rural livelihoods. *World Dev.* **27**, 2021–2044 (1999).
16. Hainmueller, J. Entropy balancing for Causal Effects: A Multivariate Reweighting Method to Produce Balanced Samples in Observational Studies. *Polit. Anal.* **20**, 25–46 (2012).
17. Rubin, D. B. An Overview of Multiple Imputation. *Proc. Surv. Res. methods Sect. Am. Stat. Assoc.* (1988).
18. Wineman, A. & Jayne, T. S. Land Prices Heading Skyward? An Analysis of Farmland Values across Tanzania. *Appl. Econ. Perspect. Policy* **40**, 187–214 (2018).
19. Alkire, S., Roche, J. M., Santos, M. E. & Seth, S. Multidimensional Poverty Index 2011: Brief

Methodological Note. *Oxford Poverty Hum. Dev. Initiat. Publ.* 1–14 (2011).

20. Imai, K., Keele, L. & Yamamoto, T. Identification, inference and sensitivity analysis for causal mediation effects. *Stat. Sci.* **25**, 51–71 (2010).

21. Baron, R. & Kenny, D. A. The Moderator-Mediator Variable Distinction in Social Psychological Research: Conceptual, Strategic, and Statistical Considerations. *J. Personal. Soc. Psychol.* **51**, 1173–1182 (1986).

22. White, H. Theory-based impact evaluation: principles and practice. *J. Dev. Eff.* (2009) doi:10.1080/19439340903114628.

23. Raphael, K. Recall bias: A proposal for assessment and control. *Int. J. Epidemiol.* **16**, 167–170 (1987).

24. Stuart, E. A. Matching Methods for causal inference: A reivew and a look forward. *Stat. Sci.* **25**, 1–21 (2010).

25. Farr, T. G. *et al.* The Shuttle Radar Topography Mission. *Rev. Geophys.* **45**, (2007).

26. Schaetzl, R. J., Krist, F. J. & Miller, B. A. A taxonomically based ordinal estimate of soil productivity for landscape-scale analyses. *Soil Sci.* (2012) doi:10.1097/SS.0b013e3182446c88.

27. Open Street Map Planet File. https://planet.openstreetmap.org/.

28. IUCN & UNEP. The World Database on Protected Areas. *The World Database on Protected Areas (WDPA)* (2011) doi:www.protectedplanet.net.

29. Fick, S. E. & Hijmans, R. J. WorldClim 2: new 1-km spatial resolution climate surfaces for global land areas. *Int. J. Climatol.* **37**, 4302–4315 (2017).

30. Hansen, M. C. *et al.* High-resolution global maps of 21st-century forest cover change. *Science* **342**, 850–3 (2013).

31. *WorldPop (www.worldpop.org)*. https://dx.doi.org/10.5258/SOTON/WP00645%0A%0A (2018).
